# Regulation of *Il6* expression by single CpG methylation in downstream of *Il6* transcription initiation site

**DOI:** 10.1016/j.isci.2022.104118

**Published:** 2022-03-18

**Authors:** Benedict Shi Xiang Lian, Takumi Kawasaki, Norisuke Kano, Daisuke Ori, Moe Ikegawa, Ayako Isotani, Taro Kawai

**Affiliations:** 1 Laboratory of Molecular Immunobiology, Division of Biological Science, Graduate School of Science and Technology, Nara Institute of Science and Technology (NAIST), Nara 630-0192, Japan; 2 Laboratory of Organ Developmental Engineering, Division of Biological Science, Graduate School of Science and Technology, Nara Institute of Science and Technology (NAIST), Nara 630-0192, Japan

**Keywords:** Immunology, Molecular biology

## Abstract

The innate immune system is an immediate defense against infectious pathogens by the production of inflammatory cytokines and other mediators. Deficiencies of epigenetic regulatory enzymes, such as *Tet1* and *Dnmt1*, cause dysregulation of cytokine expression. However, it is unclear if DNA methylation at a single CpG dinucleotide in a specific gene locus can regulate gene expression. In this study, we demonstrated that CpG+286 and CpG+348 in exon 2 of the *Il6* gene are similar in various primary mouse cells. In lipopolysaccharide-stimulated condition, hypomethylated CpG+286 promoted *Il6* expression whereas deletion of CpG+348 led to a reduction in *Il6* expression associated with enhanced CTCF binding to the *Il6* locus. Moreover, hypomethylation at CpG+286 in alveolar macrophages from aged mice led to higher *Il6* expression in response to LPS compared with young mice. Thus, DNA methylation at specific CpG dinucleotides plays an important regulatory role in *Il6* expression.

## Highlights

• TET enzymes regulate DNA methylation at *Il6* TIS region and control *Il6* expression
• DNA methylation level at CpG+286 in the *Il6* locus directly controls *Il6* expression
• Methylated CpG+348 regulates *Il6* expression and CTCF recruitment to the *Il6* locus
• Methylation of CpG+286 in alveolar macrophages controls age-related *Il6* expression

## Graphical abstract

**Figure:**
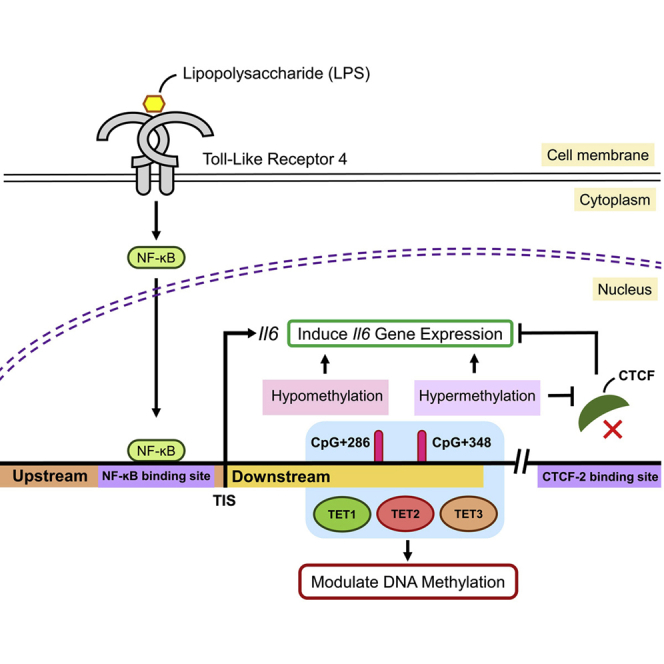

## Introduction

The innate immune system is the first defense against pathogens, protecting the host from infection and initiating the antigen-specific adaptive immune response for effective clearance of pathogens (Kawai and Akira, 2010). Macrophages and dendritic cells (DCs) play an essential role in facilitating innate immune responses to produce mediators such as proinflammatory cytokines (IL-6, IL-1β, and TNF-α) and interferons (IFN-α and IFN-β). IL-6 is a soluble mediator that exerts various pleiotropic effects on inflammation, the immune response, and hematopoiesis. *Il6* gene transcription is mainly activated by the transcription factor NF-κB via the MyD88-dependent pathway initiated by several types of Toll-like receptors (TLRs) that recognize pathogen-associated molecular patterns (Tanaka et al., 2014). Dysregulation of IL-6 production leads to chronic inflammation, autoimmune diseases, and tumorigenesis (Hirano, 2021).

DNA methylation in vertebrates involves the chemical modification of DNA by adding a methyl group (-CH_3_) at the 5-carbon position of cytosine (5mC) in the cytosine–phosphate–guanine (CpG) dinucleotide (Robertson, 2005). DNA methylation is a reversible and heritable chemical reaction, facilitated by DNA methyltransferases and DNA methyl-dioxygenase (Wu and Zhang, 2014). Two DNA methyltransferases, DNMT3a and DNMT3b, are responsible for *de novo* methylation, and DNMT1 maintains DNA methylation during cell division (Brenner and Fuks, 2006). Meanwhile, three classes of DNA demethylases, TET1, TET2, and TET3, perform DNA demethylation at designated regions in the genome (Ito et al., 2011). Several findings have reported the involvement of DNMTs and TETs in the innate immune response. siRNA knockdown of *Tet1* showed a decrease in global DNA demethylation and reduced TNF-α expression, indicating DNA hypomethylation by TET1 at the *Tnfa* gene promoter (Sun et al., 2019). Another study in *Tet1*-knockout (KO) mice showed that the loss of TET1 caused DNA hypermethylation at several CpG dinucleotides in the *Irf7* locus and many other gene loci, which contributed to the interferon and aryl hydrocarbon receptor (AhR) pathways to suppress mouse lung inflammation (Burleson et al., 2019). A vital role has also been demonstrated for TET2 in suppressing inflammation during bacterial lipopolysaccharide (LPS) stimulation in innate immune cells by recruiting HDAC2 to suppress *Il6* expression (Zhang et al., 2015; Cull et al., 2017). Meanwhile, several studies demonstrated that DNMT3b and DNMT1 are needed to regulate macrophage polarization and inflammation by inducing DNA hypermethylation at the *Pparγ* and *Klf4* gene promoters (Yang et al., 2014; Wang et al., 2016; Tang et al., 2019). Overall, these findings show that regulation of DNA methylation by epigenetic enzymes controls gene expression, contributing to innate immune responses.

Gene expression is regulated by CpG dinucleotides located around the transcription initiation site (TIS) (Saxonov et al., 2006). Loci with high frequencies of CpG dinucleotides are known as CpG islands (CGI) (Takai and Jones, 2002). CGI DNA methylation plays a regulatory role in gene expression by inducing long-term gene silencing, such as X chromosome inactivation and genomic imprinting (Hashimshony et al., 2003). In contrast, DNA methylation of single CpG dinucleotides in low CpG content regions is conserved in transcription binding motifs, and it is still unclear if this plays a regulatory role in gene expression (Yin et al., 2017). CCCTC-binding factor (CTCF) is a zinc finger protein that is highly conserved in vertebrates, with diverse regulatory roles in mediating gene expression (Kim et al., 2015). Numerous findings have demonstrated that DNA methylation of a CpG dinucleotide located in the CTCF-binding motif prevents binding of CTCF to the motif (Bell and Felsenfeld, 2000; Filippova et al., 2001; Shukla et al., 2011). CTCF also organizes the chromatin with cohesin to form topologically associating domains (TAD) that block promoter and enhancer interactions (Kentepozidou et al., 2020). Other reported functional roles of CTCF in regulating gene transcription include the recruitment of the large subunit of RNA polymerase II, and mediating alternative splicing and RNA polymerase II transcriptional pausing (Chernukhin et al., 2007; Kang and Lieberman, 2011; Shukla et al., 2011).

DNA methylation plays an essential role in the innate immune response. However, the specific mechanism by which DNA methylation regulates cytokine expression in response to LPS stimulation remains unclear. Here, we identified a functional role for DNA methylation at single CpG dinucleotides in the *Il6* gene. We found that the *Il6* locus has low CpG content and that DNA methylation of single CpG dinucleotides downstream of the TIS modulates *Il6* expression. CRISPR/deactivated Cas9 (dCas9) fused with eukaryotic DNA methyltransferases or methyl-dioxygenases has been applied to study the role of DNA methylation at the *Il6* locus. We found that *Il6* expression after LPS stimulation was controlled by DNA methylation of a single CpG dinucleotide downstream of the TIS in the *Il6* locus. Meanwhile, the loss of DNA methylation reduced recruitment of the gene insulator CTCF. Moreover, our study also revealed that alveolar macrophages (AMs) from aged mice showed significantly lower methylation levels at the single CpG dinucleotide, leading to higher *Il6* expression in response to LPS compared with young mice. Thus, DNA methylation at specific CpG dinucleotides in the *Il6* locus plays an important regulatory role in gene expression.

## Results

### DNA methylation profile of the *Il6* locus

**Figure 1:**
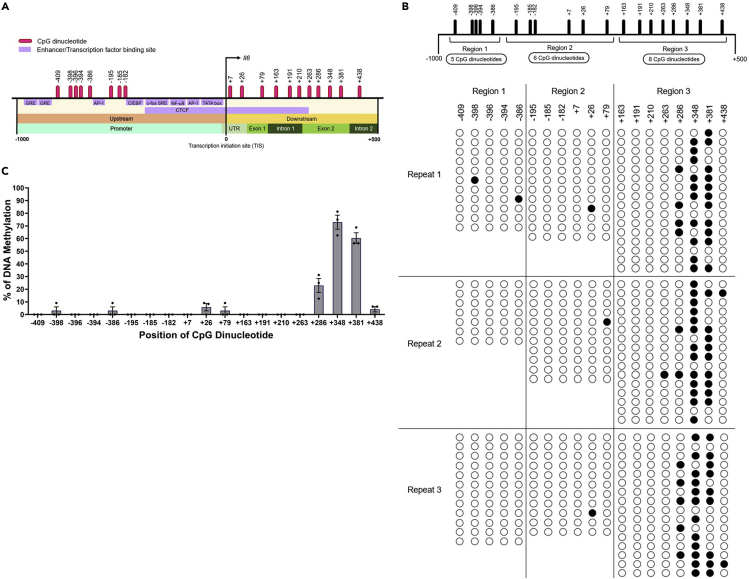
DNA methylation upstream and downstream of the TIS in the murine *Il6* locus (A) The distribution of CpG dinucleotides in the *Il6* locus. Nineteen single CpG dinucleotides are distributed around the TIS. (B) The regions upstream and downstream of the TIS were divided into three regions (regions 1, 2, and 3) for bisulfite sequencing. Filled and open circles represent the methylated and unmethylated cytosines, respectively, in the CpG dinucleotides. Each line is an individual DNA clone and each column is the CpG dinucleotide in the amplicon. (C) Bar graph showing the percentage (mean ± s.e.m) of DNA methylation in respective CpG dinucleotides.

To investigate the DNA methylation status at the TIS of *Il6*, we identified CpG dinucleotides located in the *Il6* promoter region, which is 1 kb upstream and 0.5 kb downstream of the TIS. The entire 1.5 kb of the *Il6* locus has no CpG islands, although it has 19 single CpG dinucleotides (Figure 1A). We separated the *Il6* promoter region into three regions, region 1 (−1000 b to −350 b), region 2 (−351 b to +100 b), and region 3 (+101 b to +500 b). Region one contained five single CpG dinucleotides at positions −409, −398, −396, −394, and −386. Region 2 contained six single CpG dinucleotides at positions −195, −185, −182, +7, +26, and +79. Region three contained eight single CpG dinucleotides at positions +163, +191, +210, +263, +286, +348, +381, and +438 (Figure 1B). DNA methylation in RAW264.7 cells was analyzed using bisulfite sequencing by amplifying each region with PCR, which revealed that CpG dinucleotides at the distal end of region 3 showed a higher DNA methylation rate compared with the CpG dinucleotides in regions 1 and 2 (Figure 1B). Among the 19 CpG dinucleotides, only three CpG dinucleotides at +286 (CpG+286), +348 (CpG+348), and +381 (CpG+381) in region 3 were highly methylated (20%–70%) (Figure 1C and Table S1A). These highly methylated CpG dinucleotides are located in exon 2 of the *Il6* gene (Figures 1A and 1C). Next, we examined LPS-dependent DNA methylation of the CpG dinucleotides in the *Il6* gene locus. *Il6* expression in RAW264.7 cells significantly increased from 3 to 48 h after LPS simulation and peaked at 24 h (Figure S1A), whereas the DNA methylation profile was not significantly altered at 6, 12, or 24 h after LPS stimulation (Figures S1B, S9K–S9N and Table S1G). These data indicate that DNA methylation at single CpG dinucleotides in the *Il6* gene was maintained irrespective of LPS stimulation.

### *Il6* expression and DNA methylation of CpG dinucleotides in the *Il6* locus were altered by *Tet* deficiency

**Figure 2:**
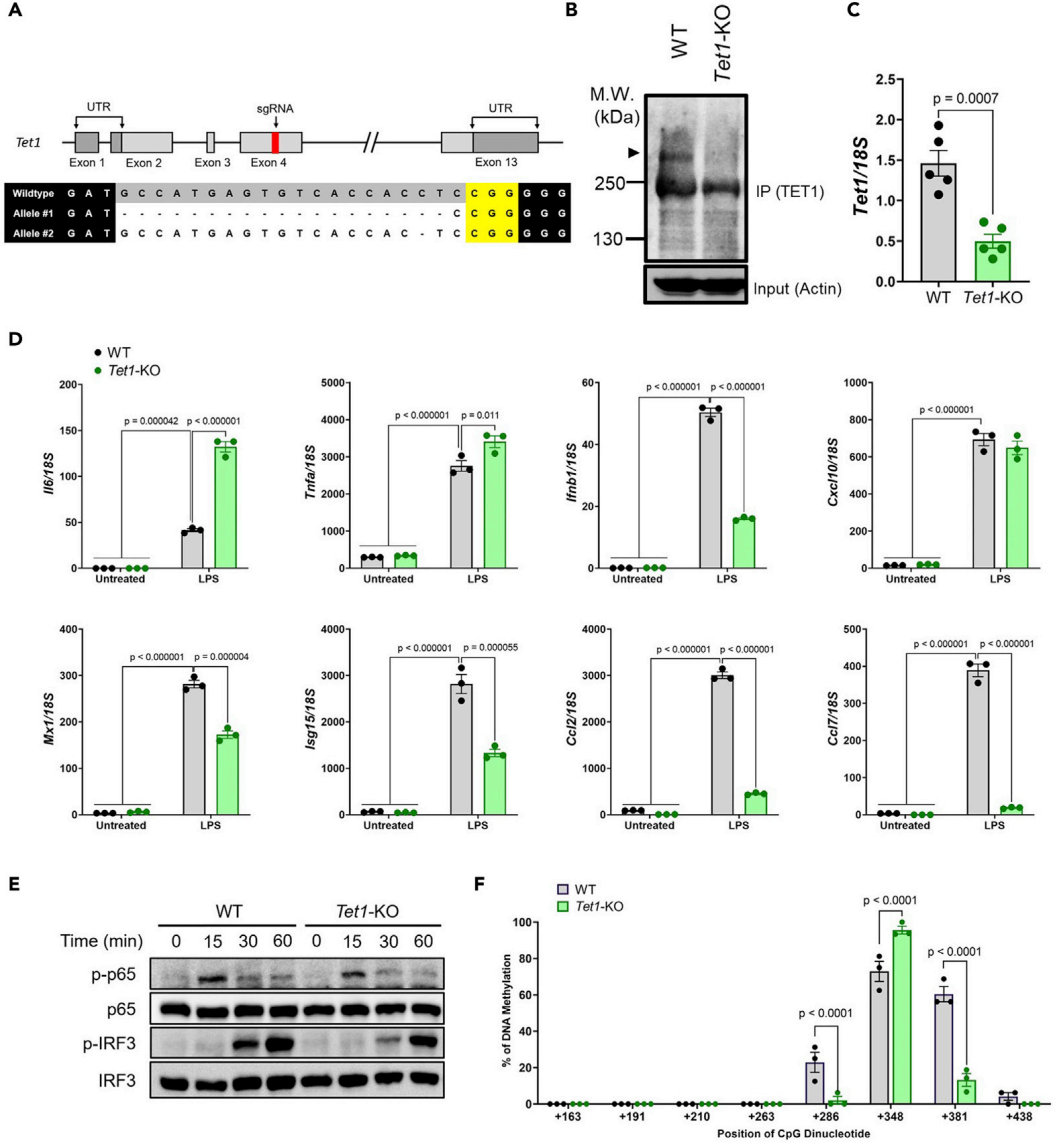
*Tet1* deficiency changed proinflammatory cytokine gene expression and DNA methylation levels at the *Il6* locus (A) Upper: schematic diagram of the *Tet1* gene. The sgRNA targeting exon 4 is highlighted in the red band. Lower: sequence of *Tet1*-KO cells. sgRNA is indicated in gray and the PAM sequence is indicated in yellow. (B) Cell lysates from WT and *Tet1*-KO cells were subjected to IP-WB with anti-TET1 antibody. Samples were blotted by anti-TET1 and anti-Actin antibodies. (C) *Tet1* expression in WT and *Tet1*-KO cells measured by RT-qPCR. (D) WT and *Tet1*-KO cells were stimulated with LPS, and expression of *Il6*, *Tnfa*, *Ifnb1*, *Cxcl10*, *Mx1*, *Isg15*, *Ccl2*, and *Ccl7* was measured by qRT-PCR (mean ± s.e.m; n = 3). (E) Cell lysates from WT and *Tet1*-KO cells after LPS stimulation were subjected to WB and were blotted with the indicated antibodies. (F) The percentage (mean ± s.e.m) of DNA methylation in respective CpG dinucleotides at the downstream region of the *Il6* locus in WT and *Tet1*-KO cells. Two-way ANOVA with Tukey’s multiple comparison test (D), unpaired two-tailed *t*-test (C), or multiple unpaired *t*-test (F).

To investigate the regulatory role of DNA methylation in the *Il6* locus, we generated *Tet1*-KO RAW264.7 cells using the CRISPR/Cas9 genome editing system with sgRNA targeting exon 4 of *Tet1* (Figure 2A). Cells with a frame-shift mutation of 19 base pairs (bps) and 1 bp deletion in each allele were selected (Figure 2A), and *Tet1* deficiency was confirmed with immunoprecipitation-Western blot (IP-WB) (Figure 2B) and qRT-PCR (Figure 2C). TET1 expression was not detected in IP-WB; therefore, *Tet1*-mutant cells could be defined as *Tet1*-KO cells (Figure 2B), and *Tet1* mRNA expression was significantly decreased in *Tet1*-KO cells (Figure 2C). Subsequently, both WT and *Tet1*-KO cells were stimulated with LPS and cytokines, and antiviral/antibacterial genes, including *Il6*, *Tnfa*, *Ifnb1*, *Cxcl10*, *Mx1*, *Isg15*, *Ccl2*, and *Ccl7*, were measured by RT-qPCR (Figure 2D). *Il6* and *Tnfa* gene expression increased significantly after LPS stimulation in *Tet1*-KO cells compared with WT cells. The *Cxcl10* gene showed comparable expression to WT cells, whereas the other cytokine genes (*Ifnb1*, *Mx1*, *Isg15*, *Ccl2*, and *Ccl7*) showed significantly reduced gene expression in *Tet1*-KO cells after LPS stimulation.

We next investigated downstream signaling pathways including NF-κB and IRF-3 activation during LPS stimulation, and found that the phosphorylation levels of NF-κB and IRF3 in WT and *Tet1*-KO cells were comparable (Figure 2E). These results suggest that *Tet1* deficiency did not alter signal transduction during LPS stimulation. We therefore proceeded to investigate the DNA methylation profile of the CpG dinucleotides in the *Il6* locus in *Tet1*-KO cells. The DNA methylation profile of the CpG dinucleotides at CpG+286 and CpG+381 showed significant hypomethylation in *Tet1*-KO cells compared with WT cells (Figure 2F). In contrast, CpG+348 showed hypermethylation in *Tet1*-KO cells (Figures 2F, S9A and Table S1B). Hence, these data suggest that modulation of the DNA methylation profile at single CpG dinucleotides at CpG+286, CpG+348, and CpG+381 by *Tet1* deficiency individually or cumulatively regulates *Il6* expression.

We also investigated the functional roles of *Tet2* and *Tet3* because both *Tet2* and *Tet3* have higher gene expression than *Tet1* in RAW264.7 cells (Figure S1C). *Tet2*-KO and *Tet3*-KO RAW264.7 cells were generated using the same strategy as *Tet1*-KO cells by targeting exon 3 for both *Tet2* and *Tet3* (Figures S2A and S3A). *Tet2*-KO and *Tet3*-KO cells demonstrated significant decreases in gene expression (Figure S2B and S3B). *Tet2*-KO cells exhibited higher *Il6* expression after LPS treatment. In contrast, LPS-treated *Tet3*-deficient cells showed a significant decrease in *Il6* expression compared with WT cells (Figures S2C and S3C). The signal transductions during LPS stimulation in both *Tet2*-KO and *Tet3-KO* cells were also comparable to WT cells (Figures S2D and S3D). *Tet2*-KO cells displayed significant hypermethylation at CpG+348 and CpG+381 compared with WT cells (Figure S2E, S9O and Table S1H). Hypermethylation was also seen at CpG+286 and CpG+381 in *Tet3*-KO cells, but CpG+348 exhibited hypomethylation in *Tet3-*KO cells compared with WT cells (Figure S3E, S9P and Table S1I). Therefore, these findings suggest that DNA methylation of CpG+286, CpG+348, and CpG+381 in the downstream region of the TIS is regulated by TET family enzymes and controls *Il6* expression during LPS stimulation.

### Hypermethylation of CpG+286 in the downstream region of the TIS reduced *Il6* expression

Although TET modulates DNA methylation in various regions of the genome, it is unclear whether DNA methylation of single CpG dinucleotides in the *Il6* locus directly regulates its gene expression. We then manipulated DNA methylation using a fusion protein of inactive Cas9 (dCas9) with DNA methyltransferase MQ1, which is a methyltransferase from the prokaryote *Mollicutes spiroplasma* (M. Sss1), strain MQ1 (dCas9-MQ1) (Lei et al., 2017). dCas9-MQ1(WT) targets CpG dinucleotides for methylation that surround a sgRNA-targeted DNA by about 300 bases upstream and downstream (Figures S4A and S4B). We first tested two sgRNAs, sgRNA1(+251) and sgRNA2(+263), which are located close to CpG+286, CpG+348, and CpG+381 (Figure S4B). *Tet1*-KO cells showed a low rate of CpG+286 and CpG+381; therefore, dCas9-MQ1(WT) with sgRNA1(+251) or sgRNA2(+263) was expressed in *Tet1*-KO cells, and the DNA methylation profile at the *Il6* locus was analyzed by bisulfite sequencing. dCas9-MQ1(WT) + sgRNA1(+251) induced robust hypermethylation at CpG+163, +191, +210, +263, +348, +381, and +438, whereas expression of dCas9-dMQ1, a mutant MQ1 with a loss of methyltransferase activity, did not enhance DNA methylation at any of these positions (Figures S4C, S9Q and Table S1J). dCas9-MQ1(WT) + sgRNA2(+263) also induced hypermethylation at these positions, except at CpG+263, which is located in the sequence targeted by sgRNA2(+263) (Figures S4D, S9R and Table S1K). Therefore, sgRNA1(+251) was selected for the subsequent experiment. Then, dCas9-MQ1(WT) or dCas9-dMQ1 with sgRNA1(+251) was expressed in RAW264.7 cells and the DNA methylation in the *Il6* locus was analyzed by bisulfite sequencing. Expression of dCas9-MQ1(WT) induced a significant increase of the DNA methylation level at CpG+7, +26, +79, +163, +191, +210, +263, and +438 (Figures S5A, S9S, S9T and Table S1L), whereas expression of dCas9-dMQ1 did not. Next, we measured *Il6* expression after LPS stimulation and found that *Il6* expression in dCas9-MQ1(WT) + sgRNA1(+251) was comparable with that in dCas9-dMQ1 + sgRNA1(+251) (Figure S5B).

**Figure 3:**
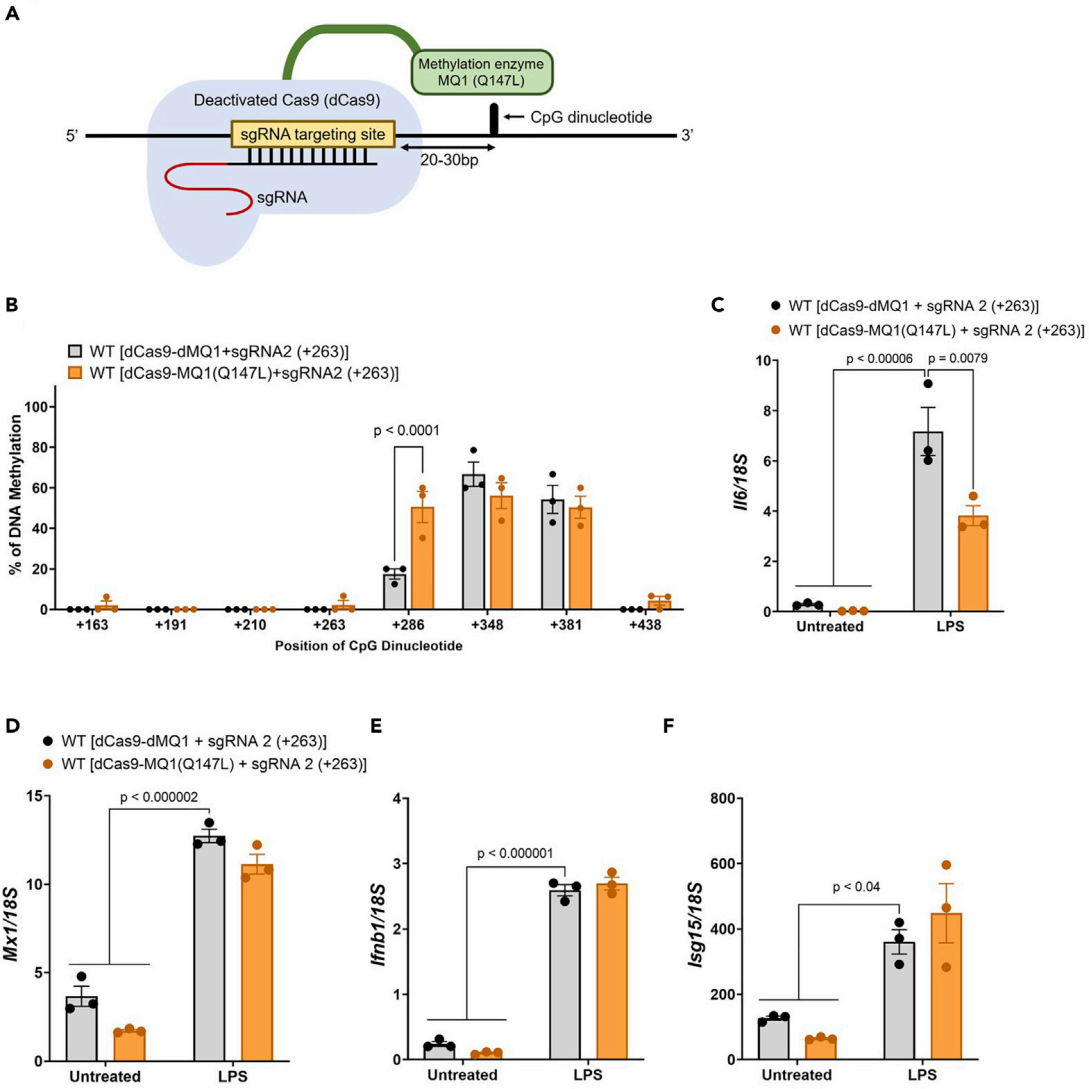
DNA hypermethylation at CpG+286 reduced *Il6* expression (A) Schematic diagram of the mechanism by which dCas9-MQ1(Q147L) facilitates hypermethylation of CpG dinucleotides. (B) The methylation profile of CpG dinucleotides in WT cells after expression of dCas9-dMQ1 + sgRNA2(+263) or dCas9-MQ1(Q147L) + sgRNA2(+263) was measured by bisulfite sequencing, and the bar graph shows the percentage of methylated CpG (mean ± s.e.m). (C–F) WT cells expressing dCas9-dMQ1 + sgRNA2(+263) or dCas9-MQ1(Q147L) + sgRNA2(+263) were stimulated with LPS, and the expression of *Il6* (C), *Mx1* (D), *Ifnb1* (E), and *Isg15* (F) were measured by qRT-PCR (mean ± s.e.m; n = 3). Multiple unpaired *t*-test (B) or two-way ANOVA with Tukey’s multiple comparison test (C, D, E, F).

**Figure 4:**
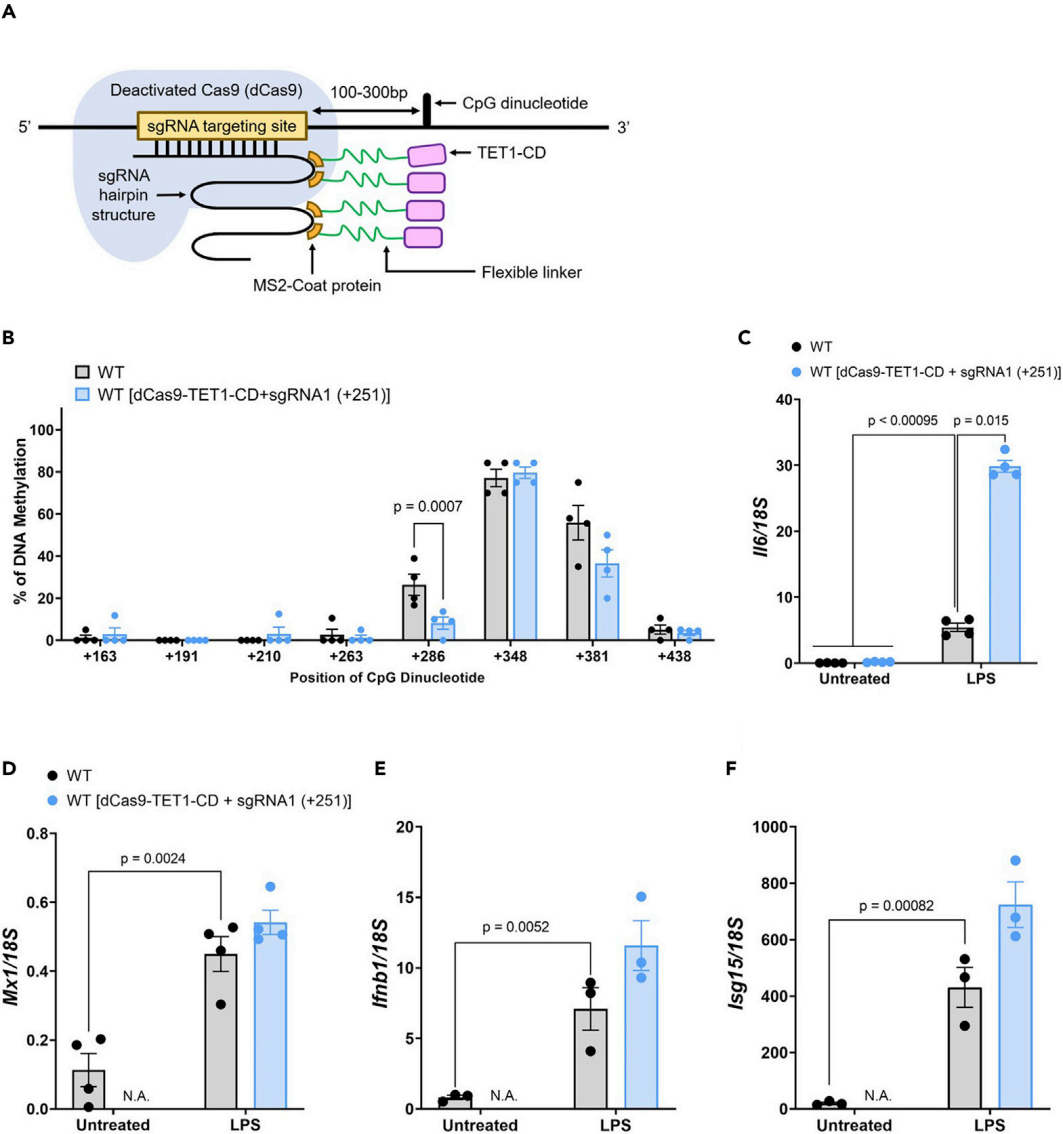
DNA demethylation at CpG+286 induced *Il6* expression (A) Schematic diagram of the mechanism by which dCas9-TET1-CD with bacteriophage MS2 coating protein facilities demethylation of the CpG dinucleotide. (B) The methylation profile of CpG dinucleotides in RAW264.7 cells after expression of dCas9-TET1-CD + sgRNA1(+251) was measured by bisulfite sequencing. Bar graph shows percentage of methylated CpG dinucleotides (mean ± s.e.m). (C–F) RAW264.7 cells expressing dCas9-TET1-CD + sgRNA1(+251) were stimulated with LPS, and the expression of *Il6* (C), *Mx1* (D), *Ifnb1* (E), and *Isg15* (F) was measured by qRT-PCR (mean ± s.e.m; n ≥ 3). Multiple unpaired *t*-test (B) or two-way ANOVA with Tukey’s multiple comparison test (C–F).

dCas9-MQ1(WT) with a sgRNA methylates a wide range of CpG dinucleotides around the targeted DNA, whereas the Q147L mutation of MQ1 (dCas9-MQ1(Q147L)) methylates a specific CpG dinucleotide located 20–30 bases downstream of the sgRNA binding site (Figure 3A) (Lei et al., 2017). RAW264.7 cells were expressed with dCas9-dMQ1 + sgRNA2(+263) or dCas9-MQ1(Q147L) + sgRNA2(+263) and the methylation profile of the *Il6*, *Mx1*, and *Ifnb* locus was measured by bisulfite sequencing analysis. We found that the methylation level of CpG+286 at region 3 in the *Il6* locus was enhanced by the expression of dCas9-MQ1(Q147L) + sgRNA2(+263), whereas CpG dinucleotides at other positions did not show enhanced levels (Figure 3B). We also measured DNA methylation in region 3′ (+42 b to +403 b) which contained CpG+79, +163, +191, +210, +263, +286, +348, and +381 and obtained similar results with the bisulfite sequence of region 3 (Figures S6A, S9U, S9V, and Table S1M). In addition, methylation level of *Mx1* and *Ifnb* locus was not altered by the expression of dCas9-MQ1(Q147L) + sgRNA2(+263) (Figures S6B, S6C, S9W–S9Z, Table S1N, and S1O). *Il6* expression after LPS stimulation was reduced by expression of dCas9-MQ1(Q147L) + sgRNA2(+263) compared with dCas9-dMQ1 + sgRNA2(+263) (Figure 3C). On the contrary, dCas9-MQ1(Q147L) expression did not alter *Mx1*, *Ifnb1*, or *Isg15* expression (Figures 4D–4F). These results demonstrate that DNA methylation at +286 inhibits *Il6* expression. Conversely, the expression of dCas9-MQ1(Q147L) + sgRNA2(+263) in *Tet1*-KO did not reduce the *Il6* expression after the LPS stimulation, despite the methylation level was significantly enhanced in CpG+286 (Figures S6D, S6E, S9AA, AB and Table S1P).

### Hypomethylation of CpG+286 increased *Il6* expression

To further study the regulatory role of DNA methylation at CpG+286 in the *Il6* locus on *Il6* expression, we expressed a fusion protein of inactive Cas9 (dCas9) with the TET1 catalytic domain (CD) tethered by bacteriophage MS2-coating protein (dCas9-TET1-CD) (Xu et al., 2016). dCas9-TET1-CD demethylates CpG dinucleotides at a specific locus 100–300 bases downstream of the targeted sgRNA (Figure 4A) (Xu et al., 2016). dCas9-TET1-CD + sgRNA1(+251) was expressed in RAW264.7 cells and CpG methylation in the *Il6*, *Mx1*, and *Ifnb* locus was analyzed by bisulfite sequencing. We found that the methylation level of CpG+286 was reduced by the expression of dCas9-TET1-CD + sgRNA1(+251), but methylation at CpG+348 and CpG+381 was not altered (Figures 4B, S9D, S9E, and Table S1D) (Figures S7A, S9AC, AD, and Table S1Q). In addition, methylation level of *Mx1* and *Ifnb* locus was not changed by the expression of dCas9-TET1-CD + sgRNA1(+251) (Figures S7B, S7C, S9AE–S9AH, Table S1R, and S1S). Furthermore, *Il6* expression after LPS stimulation was increased by the expression of dCas9-TET1-CD + sgRNA1(+251), whereas the expression of *Mx1*, *Ifnb1*, and *Isg15* was not altered (Figures 4C–4F). These findings indicate that demethylation of the CpG dinucleotide at CpG+286 promotes *Il6* expression after LPS stimulation.

### Deletion of CpG+348 reduced *Il6* gene expression but recruited higher CTCF binding

**Figure 5:**
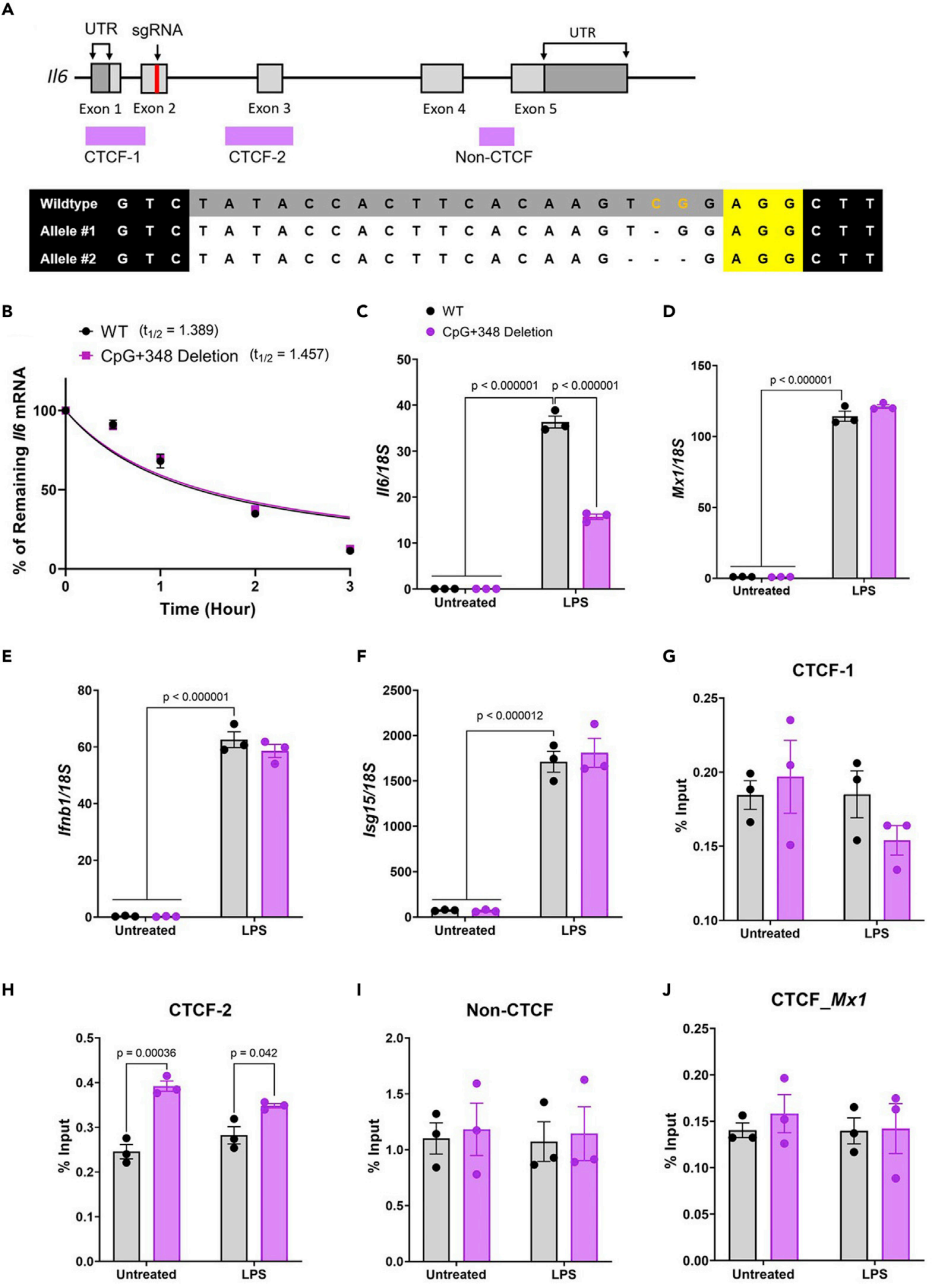
DNA methylation loss at CpG+348 reduced *Il6* expression and increased the binding of CTCF to the CTCF-2 binding site (A) Upper: schematic diagram of the *Il6* locus. The sgRNA targeting exon 2 is highlighted in the red band. Lower: sequence of CpG+348 deletion cells. The sgRNA is indicated in gray and the PAM sequence is indicated in yellow. (B) WT and CpG+348-deleted cells were treated with 5 mg/mL of actinomycin D according to the indicated time periods after LPS stimulation for 2 h. *Il6* expression was measured by qRT-PCR and fitted by exponential decay (mean ± s.e.m; n = 3). (C–F) Expression of *Il6* (C), *Mx1* (D), *Ifnb1* (E), and *Isg15* (F) in WT and CpG+348-deleted cells was measured by qRT-PCR (mean ± s.e.m; n = 3). (G–J) WT and CpG+348-deleted cells in untreated or LPS-treated cells were fixed and ChIP was performed using anti-CTCF antibody. The enrichment of CTCF at (G) CTCF-1, (H) CTCF-2, (I) Non-CTCF in *Il6* locus, and (J) CTCF region in *Mx1* locus was measured by qRT-PCR. Bar graph showing the percentage of input (mean ± s.e.m; n = 3). Unpaired two-tailed *t*-test (B) or two-way ANOVA with Tukey’s multiple comparison test (C–J).

The highest DNA methylation level in the *Il6* locus was at CpG+348; however, it could not be altered by the dCas9-MQ1 or dCas9-TET1-CD systems (Figures 3B and 4B). To reveal the functional role of CpG+348 in the regulation of *Il6* expression, we deleted CpG+348 in RAW264.7 cells using the CRISPR/Cas9 genome editing system. Deletion of CpG+348 was confirmed by sequencing analysis, and cells were selected that had a non-frame-shifted mutation in at least one allele to minimize the possibility of mRNA degradation (Figure 5A). Then, we treated both WT cells and CpG+348-deletion cells with actinomycin D after LPS stimulation and the *Il6* mRNA stability was measured. The half-life (t_1/2_) of *Il6* mRNA in WT and CpG+348-deletion cells was 1.40 and 1.46 h, respectively, indicating that the stability of *Il6* mRNA was not changed by CpG+348 deletion (Figure 5B). Then, both WT and CpG+348-deletion cells were stimulated with LPS and *Il6* expression was measured by qRT-PCR. *Il6* expression in CpG+348-deletion cells was suppressed after LPS stimulation compared with WT cells, whereas the expression of *Mx1*, *Ifnb1*, and *Isg15* did not change (Figures 5C–5F). Therefore, these results suggest that DNA methylation at CpG+348 promotes *Il6* expression after LPS stimulation.

CTCF is a transcription regulator with versatile functions that binds specific sites in the mouse genome and plays many important roles in transcriptional regulation and chromatin modeling (de Almeida et al., 2011). The *Il6* locus contains two CTCF-binding motifs (CTCF-1, -160 to +240; CTCF-2, +1439 to +1839) and CTCF recruitment has been reported (Figure 5A) (Howe et al., 2021). To further understand whether DNA methylation of CpG+348 regulates CTCF binding to the *Il6* locus, we performed a chromatin immunoprecipitation (ChIP) assay with anti-CTCF antibody. LPS-treated or untreated WT and CpG+348-deletion cells were fixed and sonicated, and then isolated DNA fragments from these cells were precipitated by anti-CTCF antibody. We selected three regions in the *Il6* locus, CTCF-1, CTCF-2, non-CTCF as a negative control and the *Mx1* locus, and the precipitated DNA was quantified by qRT-PCR (Figures 5G–5J). LPS stimulation did not alter CTCF binding to any region in either WT or CpG+348-deletion cells. We found that CpG+348 deletion increased CTCF binding to the CTCF-2 region compared with WT, but it did not alter CTCF binding to CTCF-1 region, the non-CTCF region in the *Il6* locus (Figures 5H and 5I), and CTCF region in the *Mx1* locus (Figure 5J). These findings suggest that CTCF binding to the CTCF-2 region in the *Il6* locus is regulated by DNA methylation of CpG+348.

### Age-related DNA methylation of a single CpG dinucleotide controls *Il6* expression

**Figure 6:**
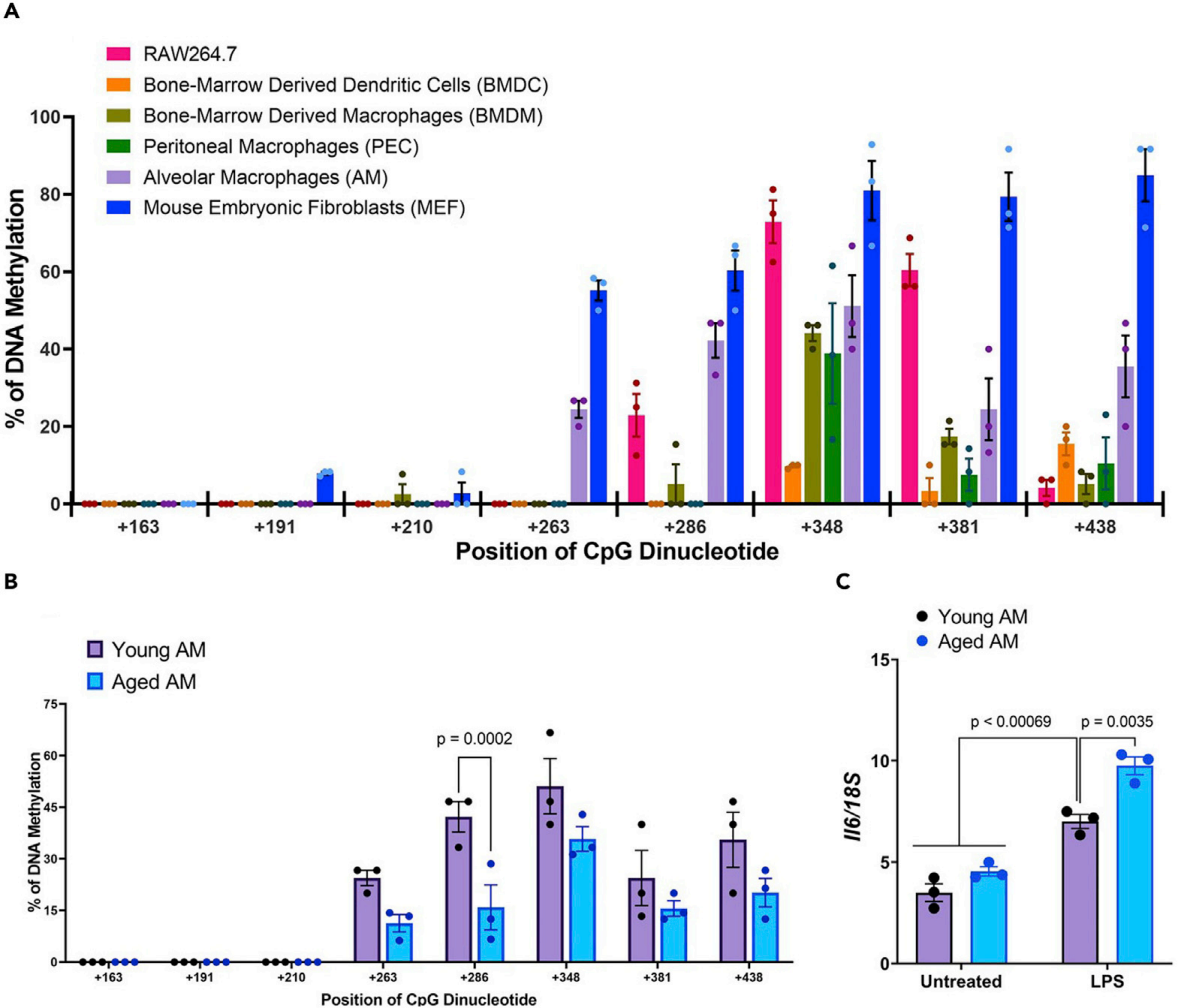
DNA methylation at CpG+286 was similar in AMs and associated with aging and *Il6* gene expression (A) The methylation profiles of various primary cell lines were measured by bisulfite sequencing. Bar graph showing the percentage of DNA methylation in CpG dinucleotides (means ± s.e.m). (B) The methylation profile of AMs from young and aged mice. (C) *Il6* expression in AMs from young and aged mice was measured before and after LPS stimulation (mean ± s.e.m; n = 3). Multiple unpaired *t*-test (B) or two-way ANOVA with Tukey’s multiple comparison test (C).

To further understand the functional role of CpG methylation, the DNA methylation profile of the *Il6* locus was analyzed in primary murine cells: bone-marrow-derived DCs (BMDCs), bone-marrow-derived macrophages (BMDMs), peritoneal macrophages (PECs), and AMs from mice aged from 8–12 weeks, and mouse embryonic fibroblast (MEFs) cells. The DNA methylation levels at CpG+348 were broadly similar in BMDMs, PECs, AMs, and MEFs, but not in BMDCs , while the DNA methylation levels at CpG+286 were high in AMs and MEFs, but not in the other primary cells (Figures 6A, S9F–S9I, and Table S1E). However, BMDCs showed a hypomethylated profile at the *Il6* locus compared with RAW264.7 cells. These results suggest that hypermethylation in CpG+286 and CpG+348 are distinctive features of macrophages, but not other cell lines.

Aging is highly associated with DNA methylation. The DNA methylation pattern in the gene promoter, the region near the TIS, the 5′-UTR, exons, and exon–intron boundaries all change significantly with age (Sziráki et al., 2018). To further investigate whether aging is associated with DNA methylation in the *Il6* locus, we isolated AMs from the lungs of young mice (8–12 weeks old) and aged mice (48–52 weeks). AMs of young mice showed higher DNA methylation levels at CpG+286 than AMs of aged mice (Figures 6B, S9I, S9J and Table S1F), whereas *Il6* expression in AMs from young mice showed lower expression levels than AMs from aged mice after LPS stimulation (Figure 6C). Then, we differentiated BMDMs from the bone marrow of young and aged mice. In contrast, BMDMs from young mice showed higher DNA methylation at CpG+348 than BMDMs from aged mice (Figures S8A, S9AI, S9AJ, and Table S1T), and BMDMs of young mice showed higher *Il6* expression than BMDMs of aged mice (Figure S8B). Therefore, these findings suggest that regulation of DNA methylation at a single CpG dinucleotide in the *Il6* locus controls *Il6* expression.

## Discussion

We found that the regions downstream and upstream of the *Il6* TIS contained 19 single CpG dinucleotides, with no CGI. Of these, only 3–5 CpG dinucleotides are methylated in RAW264.7 cells and other primary cells, which are located in exon 2 of *Il6*, downstream of the TIS. The hypermethylation or hypomethylation of CpG+286 by a CRISPR/dCas9-based system showed that hypermethylation of CpG+286 specifically suppresses *Il6* expression. Deletion of CpG+348, which shows the highest DNA methylation level in the *Il6* locus in RAW264.7 cells, showed suppression of *Il6* expression and recruitment of CTCF. These results suggest that DNA methylation of CpG+348 inhibits recruitment of CTCF, which follows suppression of *Il6* expression. In addition, we compared DNA methylation of AMs in aged and young mice and found that DNA methylation at CpG+286 was suppressed in AMs of aged mice and *Il6* expression was consequently increased. Aged mice show higher IL-6 protein expression in the alveolar lining fluid (ALF) than young mice (Moliva et al., 2014), and in agreement with this, our results showed that the DNA methylation pattern of AMs in aged mice showed hypomethylation at CpG+286 compared with young mice. We found that DNA methylation of a single CpG dinucleotide downstream of the TIS controlled gene expression, and an age-related decrease of DNA methylation levels led to changes in gene expression.

Our results indicated that a single CpG dinucleotide has a distinct regulatory role in gene expression. Expression of dCas9-MQ1(Q147L) + sgRNA2(+263) in RAW264.7 cells hypermethylated the single CpG dinucleotide at CpG+286 and decreased *Il6* expression. However, expression of dCas9-MQ1(WT) + sgRNA2(+263) in RAW264.7 cells, which induces DNA methylation at a wide range of single CpG dinucleotides in the *Il6* locus, failed to suppress *Il6* expression. Our results also showed that hypomethylation of CpG+286 increased *Il6* expression, whereas deletion of CpG+348 to mimic hypomethylation decreased *Il6* expression. Therefore, DNA methylation at a wide range of single CpG dinucleotides by dCas9-MQ1(WT) may compensate regulatory function at each CpG dinucleotide and thus did not cause gene expression changes. Our results indicated that each single CpG dinucleotide has a distinct regulatory function for gene expression.

*Tet2* and *Tet3* have higher gene expression levels compared with *Tet1* in RAW264.7 cells. Deficiency of *Tet* family genes showed a distinct distribution of altered DNA methylation in the *Il6* locus and had a regulatory role in *Il6* expression. These results indicated that DNA methylation at each CpG dinucleotide is maintained by the combination of *Tet* family enzymatic function. *Tet1* deficiency induced hypomethylation at CpG+286 and CpG+381 and hypermethylation at CpG+348, and increased *Il6* expression. As expression of dCas9-MQ1(Q147L) + sgRNA2(+263) hypermethylated CpG+286 in RAW264.7 cells, dCas9-MQ1(Q147L) + sgRNA2(+263) restored DNA methylation at CpG+286 in *Tet1*-KO cells. Expression of dCas9-MQ1(Q147L) + sgRNA2(+263) in *Tet1*-KO cells restored DNA methylation levels at CpG+286 without affecting DNA methylation at other CpG dinucleotides; however, *I16* expression was not restored. These results suggest that DNA methylation of CpG+348 and CpG+381 contributes to the regulation of *Il6* expression, or that DNA methylation at other CpG dinucleotide loci indirectly controls *Il6* expression.

DNA methylation modification by dCas9-MQ1(Q147L) or dCas9-TET1 only modified methylation at CpG+286. We have tested several gRNAs to modify the DNA methylation levels at CpG+348 and CpG+381, but none of these were successful. dCas9-MQ1 and dCas9-TET1 can directly modulate DNA methylation at CpG dinucleotides located in open chromatin regions, whereas CpG+348 and CpG+381 may be protected by chromatin-remodeling complexes or methyl-CpG binding proteins that selectively bind to methylated DNA. We deleted CpG+348 by the CRISPR/Cas9 system to mimic hypomethylation of CpG+348 and found that DNA methylation of CpG+348 inhibits recruitment of CTCF, which will induce suppression of *Il6* expression. It is unclear whether CTCF enrichment at the CTCF-2 binding site is associated with the establishment of a robust TAD boundary that leads to a stronger insulation effect on *Il6* gene transcription by blocking the interaction between enhancers and the *Il6* promoter. However, it is also possible that the CTCF enrichment might lead to histone mark modifications that silence *Il6* expression. Further study is necessary to fully determine the regulation of gene expression by DNA methylation at CpG+348, and it is important to develop tools to modulate DNA methylation of CpG dinucleotides protected by other proteins or chromatin structure. The CTCF-1 binding site spans the TIS region that contains several transcription factor binding sites, which possibly play vital roles in *Il6* transcriptional regulation. As proposed by Chernukhin et al. (2007), CTCF may recruit RNA polymerase II to the gene promoter of the *Il6* gene locus, which is also a CTCF-binding site, to initiate gene transcription in response to LPS stimulation. Hypomethylation at CpG+286 favors the recruitment of CTCF to the CTCF-1-binding site, which further promotes *Il6* expression. Overall, we propose that CTCF enrichment at the CTCF-1 and CTCF-2-binding sites might exert different functional properties in modulating *Il6* gene transcription. The correlation between CTCF recruitment and the DNA methylation level at CpG+286 and CpG+348 should be clarified in future.

In summary, we found that DNA methylation of a single CpG dinucleotide in the downstream region of the TIS regulates gene expression by modulating DNA-binding proteins. The DNA methylation level is affected by various factors, such as age, smoking, inflammation, and diet, and alterations to the DNA methylation cause changes of gene expression. It is still unclear whether this change in DNA methylation is beneficial. However, some recent reports have proposed that several CpG dinucleotides can be used as biomarkers to predict treatment outcomes and illness severity for many diseases, including cancer, pediatric diseases, autoimmune diseases, and even the recent COVID-19 (Nile et al., 2008; Claus et al., 2012; Shanthikumar et al., 2020; de Moura et al., 2021). Furthermore, a single CpG dinucleotide has a greater advantage in targeted therapy for suppressing gene expression in cells, as it has high specificity and creates fewer off-target effects. Further study is needed to determine the regulatory role of DNA methylation in gene expression and the regulation of physiological outputs by changes in DNA methylation.

### Limitations of the study

In this study, we modulated DNA methylation of single CpG dinucleotide by CRISPR/Cas9-based system. CpG+286 in the *Il6* locus was modified by the method, however, CpG+348 and CpG+381 were not. CRISPR/Cas9-based DNA methylation system has the limitation of modification in the specific CpG site; therefore, further development of the technology for DNA methylation is necessary. We proposed that *Il6* gene expression is controlled by a single DNA methylation which regulates binding of CTCF to the gene locus. Further studies are needed to clarify whether the other genes are also controlled by the DNA methylation of single CpG nucleotide in the downstream of TIS.

## STAR★Methods

### Key resources table

**Table:** 

REAGENT or RESOURCE	SOURCE	IDENTIFIER
**Antibodies**		

Mouse anti-phospho-IRF3 (Ser396)	Cell Signaling Technology	4D4G; RRID: AB_823547
Mouse anti-IRF3	Cell Signaling Technology	D83B9; RRID: AB_1904036
Mouse anti-phosphor-p65 (Ser536)	Cell Signaling Technology	93H1; RRID: AB_10827881
Mouse anti-p65	Cell Signaling Technology	D14E12; RRID: AB_10859369
Rabbit anti-TET1	Gene Tex	GTX124207; RRID: AB_11176491
Rat anti-TET1	EMD Millipore	MABE1144; RRID: AB_2910103
Mouse anti-actin	Santa Cruz	sc-47778; RRID: AB_2714189
Rabbit anti-CTCF	EMD Millipore	07-729; RRID: AB_441965
HRP-conjugated anti-rabbit	Sigma	A0545; RRID: AB_257896
HRP-conjugated anti-mouse	Sigma	A4416; RRID: AB_258167
APC anti mouse CD11c	BD Biosciences	550261; RRID: AB_398460
PE anti mouse Siglec-F	BD Biosciences	552126; RRID: AB_394341

**Bacterial and virus strains**		

Competent Quick DH5-alpha	Toyobo	DNA-913F
One Shot™ TOP10 Chemically Competent *E. coli*	ThermoFisher Scientific	C404010

**Chemicals, peptides, and recombinant proteins**		

TaKaRa ExTaq HS	TaKaRa	RR006A
pCR2.1-TOPO-TA cloning kit	Invitrogen	45-0641
KOD FX Polymerase	TaKaRa	KFX-101
ReverTra Ace Synthesis	Toyobo	TRT-101
T4 polynucletidepolynucleotide Kinase	New England Biolabs	M02015
BbsI	Thermo Scientific	ER1011
Ligation Convenience Kit	Nippon Gene	319-05961
Puromycin	Invivogen	Anti-pr-1
Hygromycin B Gold	Invivogen	Ant-hg-1
Actinomycin D	Sigma	A9415
Protein A Sepharose 4 Fast flow	Cytiva	17528001
GM-CSF	Pepro Tech	315-03
M-CSF	Pepro Tech	315-02
Collagenase	Fujifilm	032-22364
DNase	Promega	M610A
Brewer thioglycollate medium	Sigma	B2551-500G

**Deposited data**		

Supplementary Data	This paper	N/A

**Critical commercial assays**		

EZ DNA Methylation Kit	Zymo Research	D5002
RNA Extraction Kit	Zymo Research	R1035
Fast Gene Plasmid Mini Kit	Nippon Genetics	FG-90502
Nucleo Bond Xtra Midi Kit	TaKaRa	U0410C
Neon Transfection System	Invitrogen	MPK10096

**Experimental models: Cell lines**		

RAW264.7 cells	ATCC	TIB-71
HEK293T cells	ATCC	CRL-3216

**Experimental models: Organisms/strains**		

Mouse C57BL/6	CLEA Japan	N/A
Bone marrow derived macrophage from C57BL/6 mice	This paper	N/A
Bone marrow derived dendritic cells from C57BL/6 mice	This paper	N/A
Peritoneal macrophage from C57BL/6 mice	This paper	N/A
Alveolar macrophage from C57BL/6 mice	This paper	N/A

**Oligonucleotides**		

*Tet1*-KO Genotyping Primer Forward 5’-GTCAAGTCAAAGGAAGCTGTGATGC-3’	This paper	N/A
*Tet1*-KO Genotyping Primer Reverse 5’-AACTGTTAAGCAGACTAGCCGTTTTCTG-3’	This paper	N/A
*Tet2*-KO Genotyping Primer Forward 5’-ATTCCAGGATCACACAGGAAGC-3’	This paper	N/A
*Tet2*-KO Genotyping Primer Reverse 5’-TGGACTCTCATGACTGCTCTGG-3’	This paper	N/A
*Tet3*-KO Genotyping Primer Forward 5’-AAGGCCTCCTGACAAACCACC-3’	This paper	N/A
*Tet3*-KO Genotyping Primer Reverse 5’-TCAGGTTCTTGAATGGGCACC-3’	This paper	N/A
CpG+348 Genotyping Primer Forward 5’-AAGTAAGTGAAGGCAGTTCCTTGC-3’	This paper	N/A
CpG+348 Genotyping Primer Reverse 5’-TCACATTCTGTATCTTCCAGACAGG-3’	This paper	N/A

**Recombinant DNA**		

pX330-U6-Chimeric_BB-CBhhSpCas9	Addgene	#42230
pcDNA3.1-dCas9-MQ1(WT)-EGFP	Addgene	#89633
pcDNA3.1-dCas9-dMQ1-EGFP	Addgene	#89637
pcDNA3.1-dCas9-MQ1(Q147L)-EGFP	Addgene	#89634
pdCas9-TET1-CD	Addgene	#83340
pcDNA3.1-MS2-Tet1-CD	Addgene	#83341

**Software and algorithms**		

*Il6* gene TIS	Database of Transcription Start Sites (DBTSS)	https://dbtss.hgc.jp/
Transcription factor-binding site at upstream of TIS	Baccam et al. (2003)	N/A
CGI prediction and location of single CpG dinucleotide	MethPrimer 2.0 online database program	http://www.urogene.org/cgi-bin/methprimer2/MethPrimer.cgi
CTCF binding sites	Ensembl UCSC	https://asia.ensembl.org/Mus_ musculus/Info/Index https://genome.ucsc.edu/
CRISPR single guide RNA	CHOPCHOP	https://chopchop.cbu.uib.no/
Graph drawing and statistical analysis	GraphPad Prism 9	N/A

### Resource availability

#### Lead contact

Further information and requests for resources and reagents should be directed to and will be fulfilled by the Lead Contact Taro Kawai (tarokawai@bs.naist.jp).

#### Materials availability

This study did not generate new unique reagents.

### Experimental model and subject details

#### Mice

C57BL/6 mice (CLEA Japan) were bred and maintained in the specific pathogen-free animal facility. Young male mice (8–12 weeks old) and aged male mice (48–52 weeks) were used for AMs isolation. The animal maintenance and experiments performed for this study were approved by the Committee of Animal Research at Nara Institute of Science and Technology. All methods were performed based on the Policy on the Care and Use of Laboratory Animals at Nara Institute of Science and Technology.

#### Cells and reagents

RAW264.7 cells, a macrophage-like cell line from *Mus musculus*, were cultured in DMEM (Nacalai Tesque) supplemented with 10% heat-inactivated fetal bovine serum (FBS) (Life Technologies). Murine bone marrow cells were treated with 10 ng/mL M-CSF (Proteo Tech) (for BMDMs) or 10 ng/mL GM-CSF (Proteo Tech) (for BMDCs), and incubated for differentiation for 7 to 8 days at 37°C and 5% CO_2_ with RPMI 1640 (Nacalai Tesque) containing 100 units/mL penicillin, 100 g/mL streptomycin (Nacalai Tesque), 2-mercaptoethanol and 10% FBS. Brewer thioglycollate medium at 3% (w/v) was pre-injected into the peritoneal cavity of mice for 3–5 days, and 5 mL of PBS containing 3% FBS was injected to collect the peritoneal fluids. The cells obtained from the peritoneal fluids were used as primary PECs. Lungs from mice were isolated and treated with collagenase (Fujifilm 032-22364) and DNase (Promega M610A) for 30 min in RPMI 1640 medium. CD11c and Siglec F positive cells were isolated by FACS Aria II (BD Bioscience) and were used as primary AMs.

#### KO cells

To generate gene KO or CpG dinucleotide deletion using RAW264.7 cells, guide sequences located in exon 4 of *Tet1* and exon 3 of *Tet2* and *Tet3* were subcloned into pX330-U6-Chimeric_BB-CBhhSpCas9 (42230, Addgene) expressing Cas9 and gRNA: *Tet1*, sense 5′-CACCGCCATGAGTGTCACCACCTC-3′; *Tet2*, antisense 5′-CACCGAGTTCGGTTGCTTCGGTTG-3′; *Tet3*, sense 5′- CACCATTTGCACCTAGTCCCTCCG-3′; CpG+348, sense 5′- CCACTATACCACTTCACAAGTCGG-3′. The gRNAs targeting exon 3 of both *Tet2* and *Tet3* have no potential off-target sites (Table S2). The gRNA for *Tet1* and CpG+348 deletion have a few potentials off-target sites, but no mutation was found at the off-target sites in *Tet1*-KO and CpG+348-deletion cells (data not shown). Genomic regions containing guide sequences were amplified from the genome of RAW264.7 cells and inserted into the pCAG EGxxFP plasmid, acting as a reporter for genome editing. Then these plasmids at a concentration of 500 ng/μL were electroporated into RAW264.7 cells by NEON (Invitrogen) at 1680 V, 20 ms, and 1 pulse. GFP-positive cells were sorted by a BD FACS Aria (BD Bioscience). Cells were cultured for 2 weeks until cellular density reached 70%. Then, cells were transferred to 24-well plates and DNA was isolated for sequence analysis.

### Method details

#### RNA isolation and qRT-PCR

Total RNA from samples was isolated with TRIzol reagent (Invitrogen) and reverse transcribed with ReverTra Ace (Toyobo) according to the manufacturer’s instructions. qRT-PCR was performed with the following primers: m*Tet1*, forward 5′- GGACTTACTTAGCAAGCCTG-3′, reverse 5′-GGCCTTTTTCTTTTTGTGTACC-3′; m*Tet2*, forward 5′-CTCCTGGTGAACAAAGTCAGAATGG-3′, reverse 5′-CTAATAGCTGCCACATCAGGACC-3′; m*Tet3*, forward 5′-GTATGGAGAAAAGGGGAAAGC-3′, reverse 5′-AGGATCAAGATAACAATCACGG-3′; m*Il6*, forward 5′-GTAGCTATAGTACTCCAGAAGAC-3′, reverse 5′-ACGATGATGCACTTGCAGAA-3′; m*Tnfa*, forward 5′-CTGTAGCCCACGTCGTAGC-3′, reverse, 5′-TTGAGATCCATGCCGTTG-3′; m*Ifnb1*, forward 5′-ATGGTGGTCCGAGCAGAGAT-3′, reverse 5′-CCACCACTCATTCTGAGGCA-3′; m*Cxcl10*, forward 5′-CCTGCAGGATGATGGTCAAG-3′, reverse 5′-GAATTCTTGCTTCGGCAGTT-3′; m*Mx1*, forward 5′-GGGGAGGAAATAGAGAAAATGAT-3′, reverse 5′-GTTTACAAAGGGCTTGCTTGCT-3′; m*Isg15*, forward 5′-TGGAAAGGGTAAGACCGTCT-3′, reverse 5′-GGTGTCCGTGACTAACTCCAT-3′; m*Ccl2*, forward 5′-CTCAGCCAGATGCAGTTAACGCCC-3′, reverse 5′-GGTGCTGAAGACCTTAGGGCAGAT-3′; m*Ccl7*, forward 5′-TGAAAACCCCAACTCCAAAG-3′, reverse 5′-CATTCCTTAGGCGTGACCAT-3′; m*18S*, forward 5′-GTAACCCGTTGAACCCCATT-3′, reverse 5′-CCATCCAATCGGTAGTAGCG-3′.

#### WB assay

Cells were stimulated with LPS and lysed with RIPA buffer (50 mM Tris HCl, 150 mM NaCl, 0.5% sodium deoxycholate, 1% Nonidet P-40, 0.1% SDS). Whole-cell lysates were mixed with 2 ⋅ SDS sample buffer (1 M Tris-HCl, 10% SDS, 20% glycerol, 0.01% bromphenol blue, 0.2 M DTT) and heat-treated at 95°C for 5 min. Samples were subjected to SDS-PAGE and proteins were transferred to PVDF membrane (Bio-Rad). Transferred membranes were applied for blocking with 5% skim milk in TBST buffer (0.5 M Tris, 1.38 M NaCl, 0.027 M KCl, 0.05% Tween 20) and then were incubated with anti-phospho-IRF3 (Ser396) mouse antibody (4D4G; Cell Signaling Technology), anti-IRF3 mouse antibody (D83B9; Cell Signaling Technology), anti-phosphor-p65 (Ser536) mouse antibody (93H1; Cell Signaling Technology), or anti-p65 mouse antibody (D14E12; Cell Signaling Technology).

#### Immunoprecipitation and pull-down assay

Cells were cultured in 10 cm cell culture petri dishes (BD Falcon). The cells were lysed with IP-homo buffer (150 mM NaCl, 5 mM EDTA, 25 mM Tris-HCl pH 8.0, 0.1% NP-40). Lysates were incubated with anti-TET1 antibody (GTX124207, Gene Tex) and Protein A Sepharose beads (GE Healthcare) and were rotated at 4°C overnight. Antibody-bound Protein A beads were collected by spinning down and were washed three times with IP-homo buffer. Sample buffer was applied to Protein A beads and samples were prepared for WB against anti-TET1 antibody (MABE1144, EMD Millipore). For the loading control, cell lysates were blotted with anti-actinβ antibody (sc-47778, Santa Cruz). After incubation with HRP-conjugated secondary antibodies (Sigma) for 30 min, membranes were reacted with a luminescent reagent Western Lightning Plus-ECL (PerkinElmer), and proteins were detected by Image Quant LAS-4000 (GE Healthcare).

#### Chromatin immunoprecipitation assay

Cells were crosslinked by 1% formaldehyde in the medium for 10 min at room temperature and then quenched by adding 2.5 mL of 1.5 M glycine per 22 mL of media volume for 5 min. The collected cells were washed with PBS twice, and then resuspended in 5 mL of Lysis buffer 1 [50 mM HEPES-KOH (pH 7.5), 140 mM NaCl, 10 mM EDTA (pH 8.0), 10% (v/v) Glycerol, 0.5% (v/v) NP-40, 0.25% (v/v) Triton X-100] for 10 min on ice. The lysate was centrifuged for 5 min at 300 × g at 4°C. Then, the pellet was resuspended with 10 mL of Lysis buffer 2 [10 mM Tris-HCl (pH 8.0), 200 mM NaCl, 10 mM EDTA (pH 8.0)] for 10 min at room temperature. The lysate was centrifuged for 5 min at 500 × g at 4°C. Last, the pellet was resuspended with 3 mL of Lysis buffer 3 [10 mM Tris-HCl (pH 8.0), 100 mM NaCl, 10 mM EDTA (pH 8.0), 0.1% (w/v) Deoxycholic acid sodium, 0.01% (w/v) SDS]. The chromatin was sheared for 60 min by sonication with Covaris S220 (75 W, 20% duty factor, 1000 CPB) at 4°C. The cell lysate was centrifuged at maximum speed (>10,000 × g) for 15 min at 4°C and 10% of the supernatant was saved as input. Then, 10 μL of anti-CTCF antibody (07–729, EMD Millipore) was added to 500 μL and incubated overnight at 4°C. Next, 50 μL of Protein A Sepharose beads (GE Healthcare) were added into the antibody-cell lysate mixture, and the mixture was incubated for 1 h at 4°C. The beads were washed once with 1 mL of low-salt buffer [10 mM Tris-HCl (pH 8.0), 150 mM NaCl, 1 mM EDTA (pH 8.0), 0.5% (w/v) Deoxycholic acid sodium, 0.1% (w/v) SDS, 1% NP-40], once with 1 mL of high-salt buffer [10 mM Tris-HCl (pH 8.0), 500 mM NaCl, 1 mM EDTA (pH 8.0), 0.5% (w/v) Deoxycholic acid sodium, 0.1% (w/v) SDS, 1% NP-40], and once with 1 mL of LiCl buffer [10 mM Tris-HCl (pH 8.0), 250 mM LiCl, 1 mM EDTA (pH 8.0), 0.5% (w/v) Deoxycholic acid sodium, 0.1% (w/v) SDS, 1% NP-40], and last resuspended in 400 μL of elution buffer [10 mM Tris-HCl (pH 8.0), 300 mM NaCl, 5 mM EDTA (pH 8.0), 0.5% (w/v) SDS]. The eluted protein–DNA complex was treated with 10 mg/mL of RNaseA (Nippon Gene) at 37°C for one hour, and then reverse cross-linked with 10 mg/mL of Proteinase K (Wako) at 65°C overnight. The DNA was purified with phenol/chloroform/isoamyl alcohol. The DNA samples were then analyzed by qRT-PCR using the following primers for the *Il6* locus: CTCF-1 forward, 5′-AGCCCACCAAGAACGATAG-3′, reverse 5′-CAAGTGAGCAGATAGCACAG-3′; CTCF-2, forward 5′-GCTTCCCTTTTCTGTGTCC-3′, reverse 5′-GTTTTCTGCAAGTGCATCATC-3′; non-CTCF, forward 5′-TGGAGTTGTTAGGCATGGG-3′, reverse, 5′-GGAGAGCATTGGAAATTGGG-3′, the *Mx1* locus: forward 5′-TGTTTCCATTCTCAGCATCTC-3′, reverse, 5′-TCCACTCCTCTCCTTCTTTC-3′.

#### DNA methylation analysis

Bisulfite conversion of DNA was done using the EZ DNA methylation kit (Zymo Research) following the manufacturer’s instructions. Briefly, about 250 ng to 2 μg of DNA samples in 50 mM sodium hydroxide (NaOH) (Wako) with 1M Tris-HCl at pH 8.0 (Nacalai Tesque) were taken and prepared for bisulfite conversion with 100 μL of CT conversion reagent after heating at 37°C for 15 min. Then, the samples were incubated in the dark at 50°C for 18–20 h. The bisulfite-converted DNA samples were purified according to the manufacturer’s protocols. PCR was performed using Ex Taq HS (TaKaRa). The PCR primers for amplification were: Region 1 at the *Il6* locus, forward 5′-AGGGAGTGTGTGTTTTTGTATG-3′, reverse 5′-ACTTAAATCATCCTTAAAACTAACC-3′; Region 2, forward 5′-GAGTGTTTATGTTTTTTAGGGTTAG-3′, reverse 5′-AACCCACAATACTAACTCTCTACC-3′; Region 3, forward 5′-GTTATTGAGATTGTGAGAGAGG-3′, reverse 5′-ATCCTAAAAACCCTAAATCCCT-3′, Region 3′, forward 5′-TTTGTAAGTAAGTGAAGGTAGTTT-3′, reverse 5′-CACAACCTACCCACCTCTTTTCTC-3′; the *Mx1* locus, forward 5′- TTAGAATTAGTAGTTTAGGTATAA-3′, reverse 5′- ATACTAACTCCACCCCTTCCAAAA-3′; the *Ifnb* locus, forward 5′- AAATAGTATAGGTTATGAAGGAAG-3′, reverse 5′- CAATATAACTCTTCTACATCTTC-3′. The PCR conditions were as follows: 95°C for 2 min; 40 cycles of 95°C for 30 s, 55°C for 30 s, and 72°C for 1 min; then 7 min at 72°C. The amplified products were subjected to electrophoresis on a 2% agarose gel, and were then sub-cloned into a pCR2.1-TOPO-TA cloning vector (Invitrogen). Bisulfite conversion reactions were performed independently more than two times and the PCR fragments in pCR2.1-TOPO were sequenced at least 10 clones in each dot spot. The rate of methylation was calculated as the number of methylated cytosines divided by the number of total samples sequenced.

#### mRNA stability assay

The cells were stimulated with 100 ng/mL LPS (Sigma) for 2 h. Then, 5 mg/mL of Actinomycin D (Sigma A9415) was added to the culture medium. The total RNAs were prepared at the indicated periods. The total *Il6* mRNA was quantified by qRT-PCR.

#### Targeted DNA methylation and demethylation in CpG dinucleotides

sgRNA1(+251) (forward 5′-CCACTGCCTTCTTGGGACTGATGC-3′, reverse 5′-AAACGCATCAGTCCCAAGAAGGCA-3′) was subcloned into pcDNA3.1-dCas9-MQ1(WT)-EGFP (Addgene #89633), pcDNA3.1-dCas9-dMQ1-EGFP (Addgene #89637), and pdCas9-TET1-CD (Addgene #83340). sgRNA2(+263) (forward 5′-CCACACTGATGCTGGTGACAACCA-3′, reverse 5′-AAACTGGTTGTCACCAGCATCAGT-3′) was subcloned into pcDNA3.1-dCas9-MQ1(Q147L)-EGFP (Addgene #89634) and pcDNA3.1-dCas9-dMQ1-EGFP (Table S2). For the transfection of pcDNA3.1-dCas9-MQ1(WT)-EGFP, pcDNA3.1-dCas9-MQ1(Q147L)-EGFP and pcDNA3.1-dCas9-dMQ1-EGFP, 5 μL of plasmids at a concentration of 2–5 μg/μL were electroporated into RAW264.7 cells by NEON (Invitrogen) at 1680 V and 20 ms. The transfected cells were cultured in Penicillin/Streptomycin-supplemented DMEM media for 2 days and GFP-positive cells were sorted by a BD FACS Aria (BD Bioscience). Similarly, for the transfection of pcDNA3.1-MS2-Tet1-CD (Addgene #83341) and dCas9-TET1-CD, plasmids at a concentration of 2–5 μg/μL, which adjusted to a fixed molar ratio of 1:2.6 as suggested by Xu et al., were electroporated into RAW264.7 cells by NEON (Invitrogen) at 1680 V and 20 ms (Xu et al., 2016). The transfected cells were cultured in Penicillin/Streptomycin-supplemented DMEM media for 2 days and were selected by 250 μg/mL of hygromycin B gold (Invivogen) for 4 days. Both the FACS-sorted or hygromycin-selected cells were collected and stimulated with 100 ng/mL of LPS (Sigma) for 6 h. Total RNA was prepared from the samples by RNA Extraction Kit (Zymo Research) according to the manufacturer’s instructions.

#### Genome annotation

The TIS of *Il6* was obtained from the Database of Transcription Start Sites (DBTSS; Suzuki et al., 2018). CGI prediction and single CpG dinucleotide identification in *Il6* were performed using MethPrimer 2.0 online database program (Li and Dahiya, 2002). The CTCF binding sites in the *Il6* locus were obtained from Ensembl genome browser and USCS genome browser. The transcription factor-binding sites in the promoter region of *Il6* of *Mus musculus* were taken from Baccam et al. (2003) (Baccam et al., 2003).

### Quantification and statistical analysis

The methylation rate of a CpG dinucleotide is calculated as follow: rate of methylation = number of methylated cytosines/total number of clones sequenced. A two-tailed Student’s unpaired *t* test was used for two-group comparisons, a one-way or two-way ANOVA with Tukey’s multiple comparison test was used when three or more groups were compared. All statistical analysis were processed by the GraphPad Prism Version 9.0 software package (GraphPad Software, San Diego, CA).

## Data Availability

All data reported in this paper will be shared by corresponding authors upon request. This paper does not report original code. Any additional information required to reanalyze the data reported in this paper is available from corresponding authors upon request.

## Supplemental information

Document S1. Figures S1–S9 and Tables S2

Table S1. The summary of DNA methylation analysis from the respective figures
