# Supplementary material for: Regulation of Il6 expression by single CpG methylation in downstream of Il6 transcription initiation site
Source: iScience. 2022 Mar 18;25(4):104118. doi: 10.1016/j.isci.2022.104118 (PMC8983349; doi:10.1016/j.isci.2022.104118)
Supplement: Document S1. Figures S1–S9 and Tables S2 [file mmc1.pdf]

iScience, Volume *25*

Supplemental information

Regulation of *Il6* expression by single CpG methylation in downstream of *Il6* transcription initiation site

Benedict Shi Xiang Lian, Takumi Kawasaki, Norisuke Kano, Daisuke Ori, Moe Ikegawa, Ayako Isotani, and Taro Kawai

Supplementary Figure 1

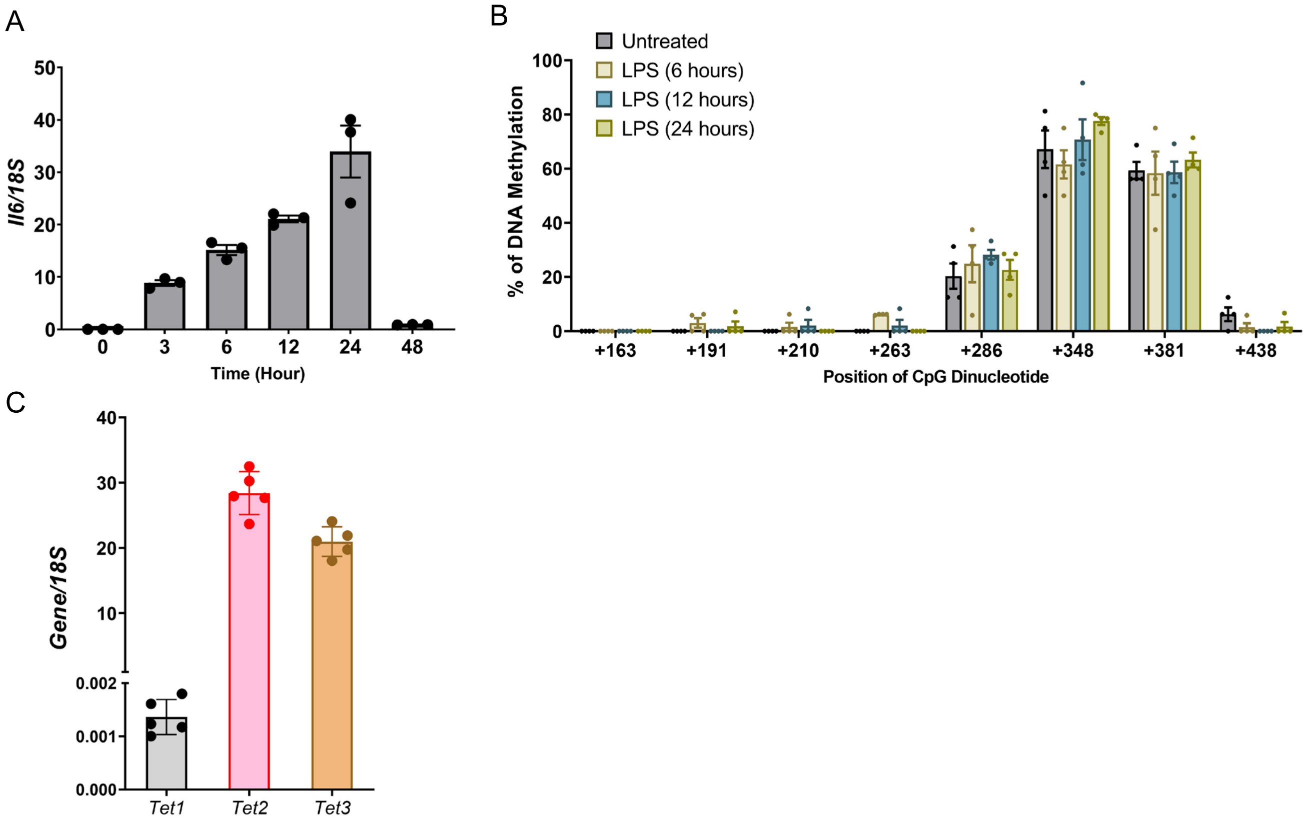

Figure S1. *Il6* expression and DNA methylation during LPS stimulation, Related to Figure 1. **(A)** RAW264.7 cells were stimulated with LPS, and the expression of *Il6* was quantified by qRT-PCR (means ± s.e.m). **(B)** The methylation profile of CpG dinucleotides was measured by bisulfite sequencing before and after 6 and 24 hours of LPS stimulation. **(C)** The expression levels of the Tet family genes, *Tet1*, *Tet2* and *Tet3*, in RAW264.7 cells quantified by qRT-PCR (means ± s.e.m).

Supplementary Figure 2

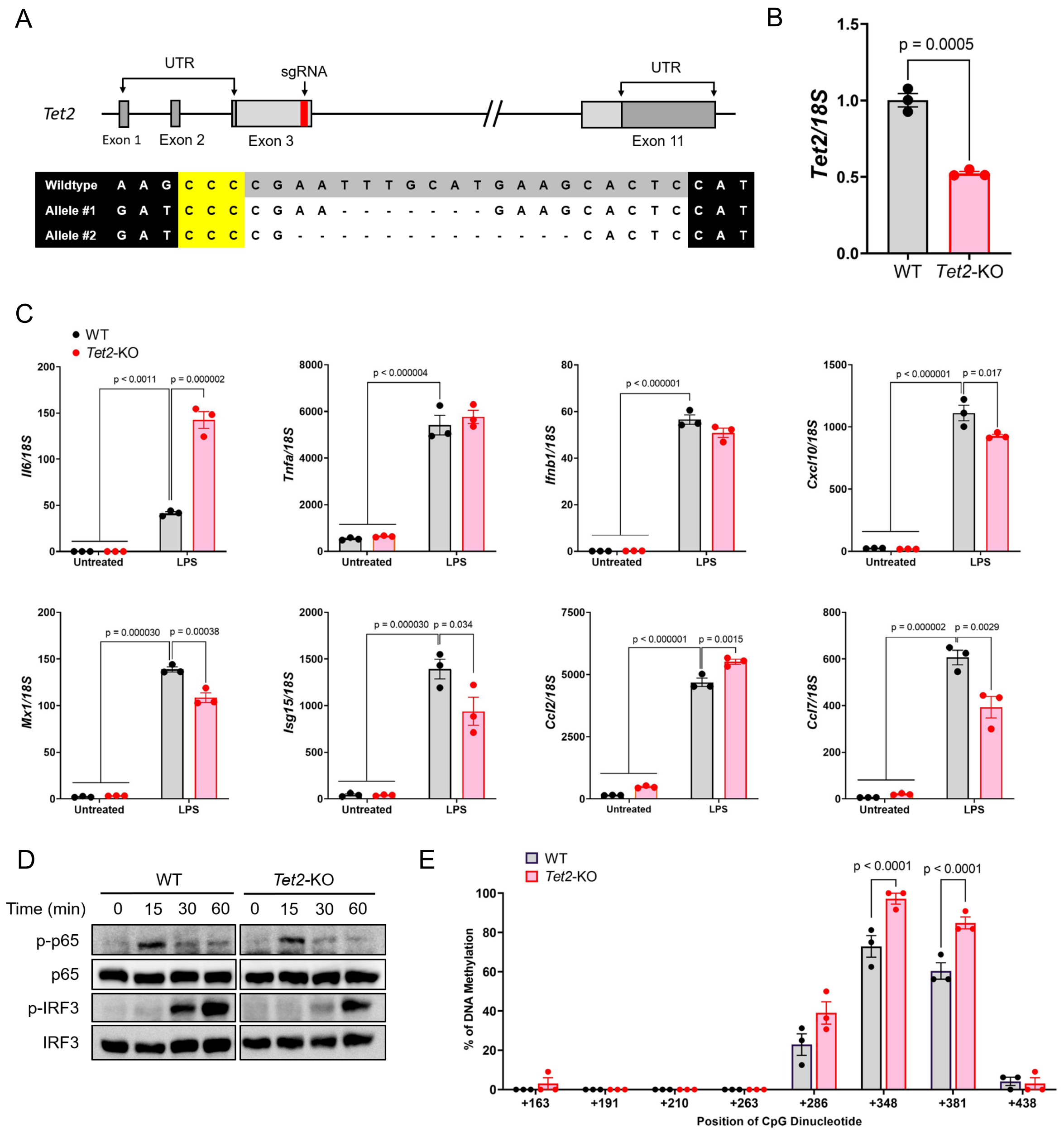

Figure S2. Proinflammatory cytokine gene expression changes in *Tet2*-KO cells are associated with the DNA methylation level, but with not the NF-κB signaling pathway, Related to Figure 2. **(A)** Upper: schematic diagram of the *Tet2* gene. The sgRNA targeting exon 3 is highlighted in the red band. Lower: sequence of *Tet2*-KO cells. The sgRNA is indicated in gray and the PAM sequence is indicated in yellow. **(B)** The gene expression of *Tet2* in control and *Tet2*-KO cells. **(C)** WT and *Tet2*-KO cells were stimulated with LPS, and expression of *Il6*, *Tnfa*, *Ifnb1*, *Cxcl10*, *Mx1*, *Isg15*, *Ccl2*, and *Ccl7* was measured by qRT-PCR (mean ± s.e.m; n = 3). **(D)** Cell lysates from WT and *Tet2*-KO cells after LPS stimulation were subjected to WB and were blotted with the antibodies indicated. **(E)** The percentage (mean ± s.e.m) of DNA methylation in respective CpG dinucleotides at the downstream region of the *Il6* gene in WT and *Tet2*-KO cells. Two-way ANOVA with Tukey's multiple comparison test (C), unpaired two-tailed t-test (B) or multiple unpaired t-test (E).

Supplementary Figure 3

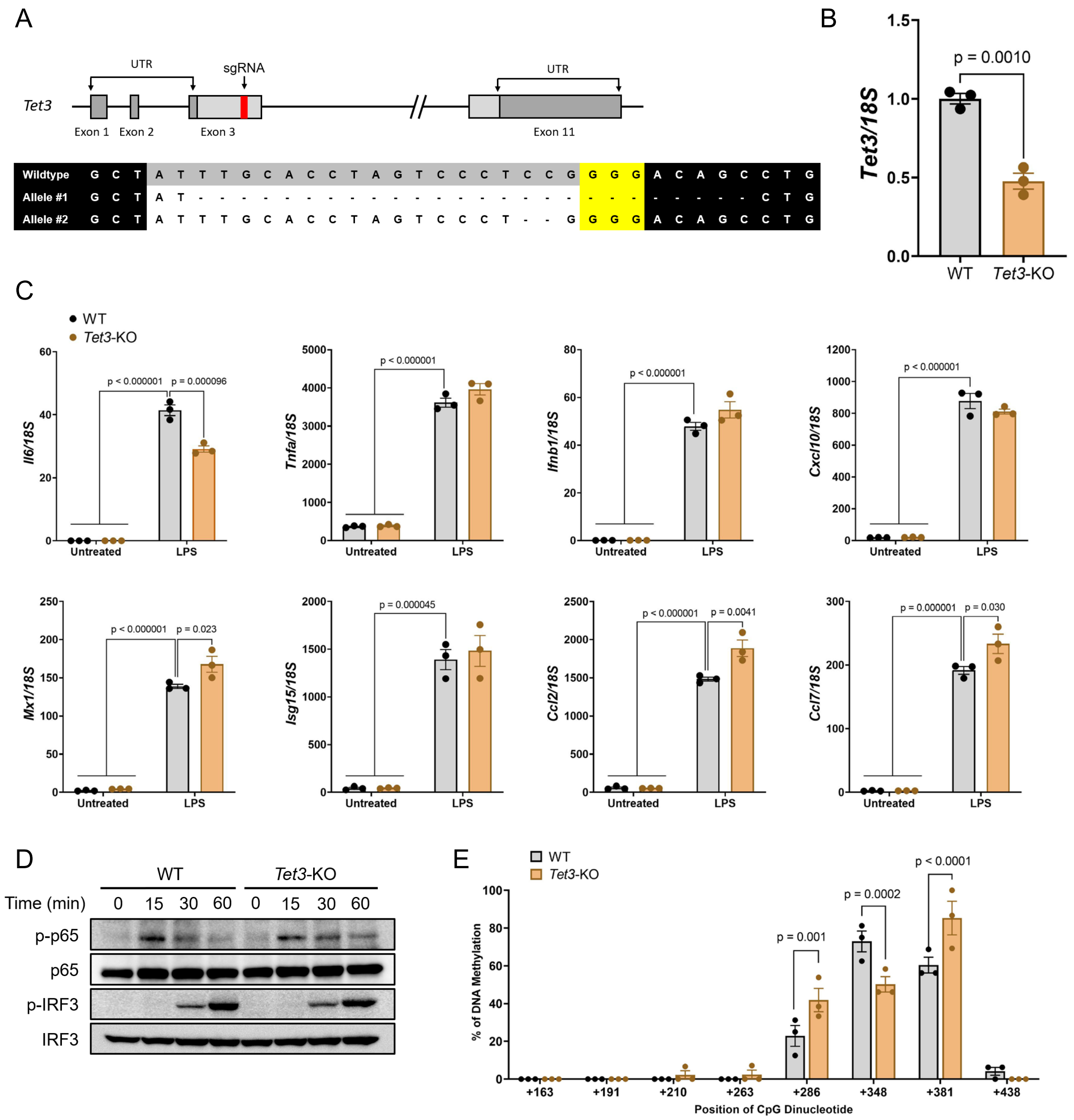

Figure S3. Proinflammatory cytokine gene expression changes in *Tet3*-KO cells are associated with the DNA methylation level, but not with the NF-κB signaling pathway, Related to Figure 2. **(A)** Upper: schematic diagram of the *Tet3* gene. The sgRNA targeting exon 3 is highlighted in the red band. Lower: sequence of *Tet3*-KO cells. The sgRNA is indicated in gray and the PAM sequence is indicated in yellow. **(B)** Gene expression of *Tet3* in control and *Tet3*-KO cells. **(C)** WT and *Tet3*-KO cells were stimulated with LPS, and expression of *Il6*, *Tnfa*, *Ifnb1*, *Cxcl10*, *Mx1*, *Isg15*, *Ccl2*, and *Ccl7* was measured by qRT-PCR (mean ± s.e.m; *n* = 3). **(D)** Cell lysates from WT and *Tet3*-KO cells after LPS stimulation were subjected to WB and were blotted with the antibodies indicated. **(E)** The percentage (mean ± s.e.m) of DNA methylation in respective CpG dinucleotides at the downstream region of the *Il6* gene in WT and *Tet3*-KO cells. Two-way ANOVA with Tukey's multiple comparison test (C), unpaired two-tailed t-test (B) or multiple unpaired t-test (E).

Supplementary Figure 4

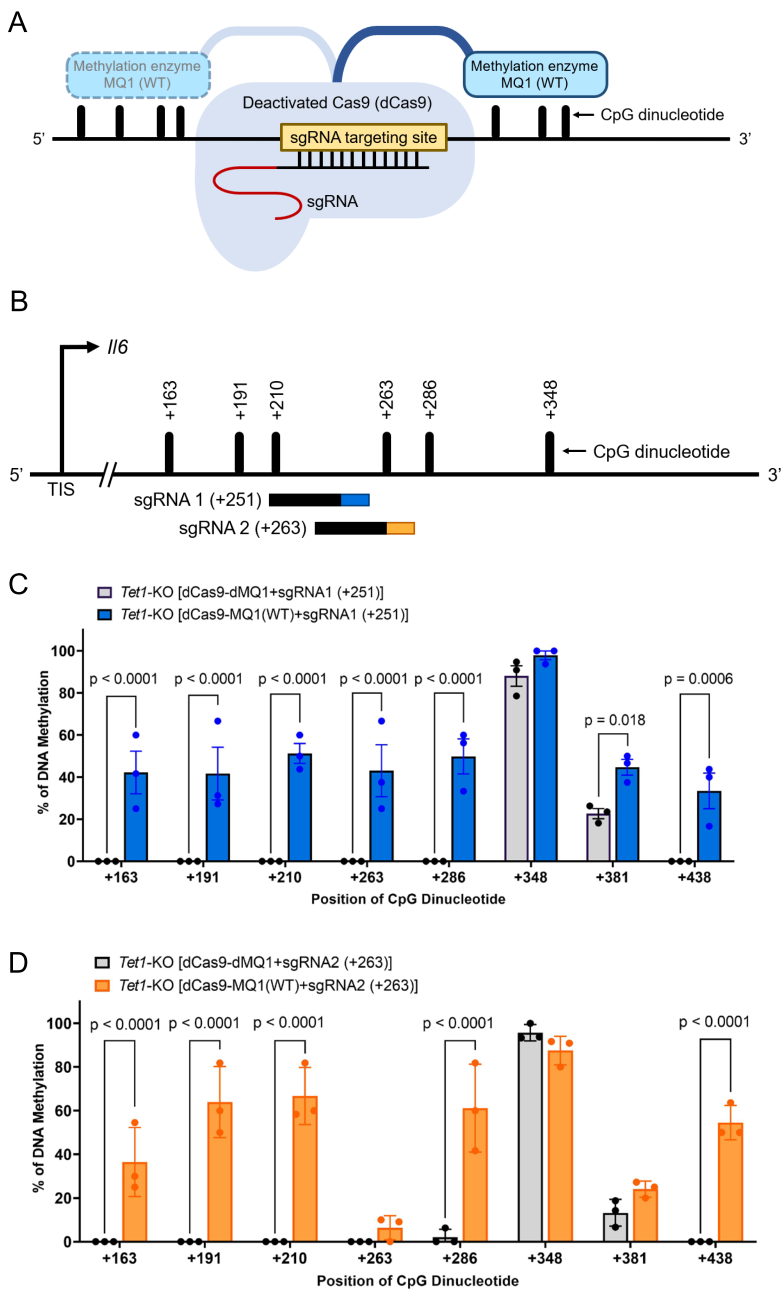

Figure S4. DNA methylation in the *Il6* locus in *Tet1*-KO cells by the expression of dCas9-MQ1(WT), Related to Figure 3. **(A)** Schematic diagram of the mechanism of dCas9-MQ1(WT) in facilitating *de novo* or hypermethylation at CpG dinucleotide(s) and **(B)** the location of sgRNA1(+251) and sgRNA2(+263) with the CpG nucleotides in the *Il6* locus. **(C, D)** The methylation profile of CpG dinucleotides in the TIS downstream region. *Tet1*-KO cells expressed dCas9-MQ1(WT) with sgRNA1(+251) or sgRNA2(+263) and the methylation profile was determined by bisulfite sequencing. Multiple unpaired t-test (C, D).

Supplementary Figure 5

A

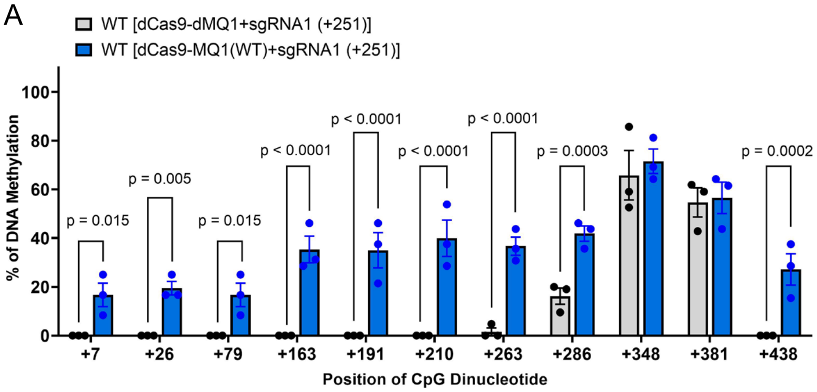

B

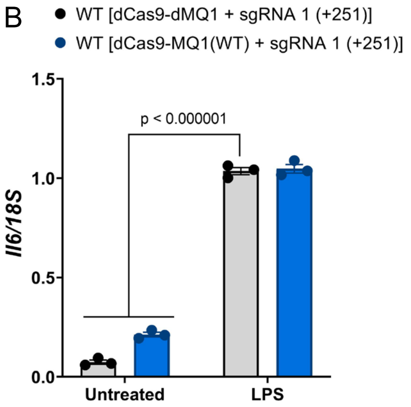

Figure S5. DNA methylation in the *Il6* locus in WT cells by the expression of dCas9-MQ1(WT), Related to Figure 3. **(A)** The methylation profile of CpG dinucleotides in WT cells after expression of dCas9-dMQ1 + sgRNA1(+251) or dCas9-MQ1(WT) + sgRNA1(+251) were measured by bisulfite sequencing, indicated as a percentage (means ± s.e.m). **(B)** WT cells that expressed dCas9-dMQ1 + sgRNA1(+251) or dCas9-MQ1(WT) + sgRNA1(+251) were stimulated with LPS, and the expression of *Il6* was measured by qRT-PCR (mean ± s.e.m; $n = 3$). Multiple unpaired t-test (A) or two-way ANOVA with Tukey's multiple comparison test (B).

Supplementary Figure 6

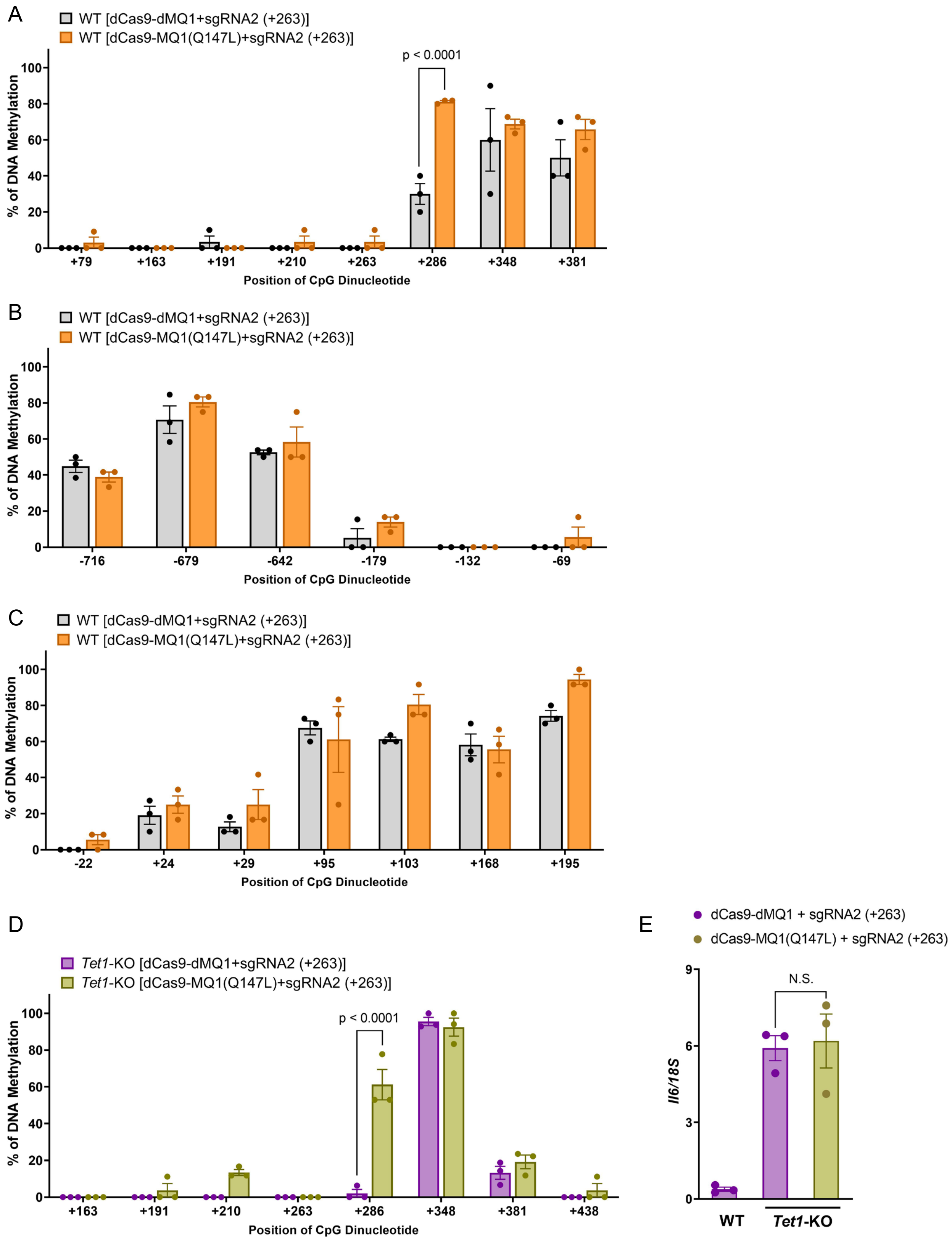

Figure S6. DNA methylation in the *Il6, Mx1* and *Ifnb* locus in WT cells by the expression of dCas9-MQ1(Q147L), Related to Figure 3. **(A-C)** The methylation profile of CpG dinucleotides in the *Il6* region 3' (A), *Mx1* (B), *Ifnb* (C) locus were measured after expression of dCas9-dMQ1 + sgRNA2(+251) or dCas9-MQ1(Q147L) + sgRNA1(+263) by bisulfite sequencing, indicated as a percentage (means ± s.e.m). **(D)** The methylation profile of CpG dinucleotides in *Tet1*-KO cells after expression of dCas9-MQ1(Q147L) + sgRNA2(+263) was measured by bisulfite sequencing, indicated as a percentage (means ± s.e.m). **(E)** WT cells that expressed dCas9-dMQ1 + sgRNA1(+263) or dCas9-MQ1(Q147L) + sgRNA1(+263) were stimulated with LPS, and the expression of *Il6* was measured by qRT-PCR (mean ± s.e.m; n = 3). Multiple unpaired t-test (A, B, C, D) or one-way ANOVA with Sidak's multiple comparison test (E).

Supplementary Figure 7

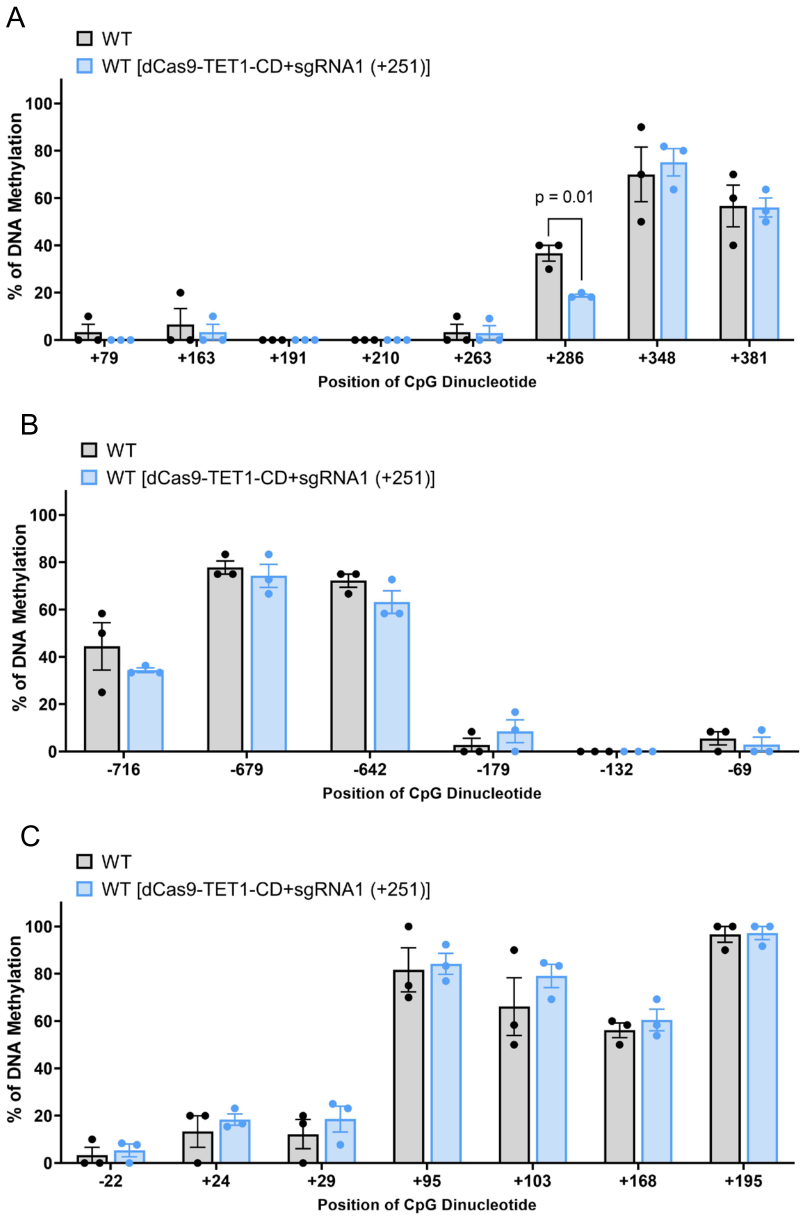

Figure S7. DNA methylation in the *Il6, Mx1* and *Ifnb* locus in WT cells by the expression of dCas9-TET-CD, Related to Figure 4. **(A-C)** The methylation profile of CpG dinucleotides in the *Il6* region 3'(A), *Mx1* (B), *Ifnb* (C) locus were measured by bisulfite sequencing after expression of dCas9-TET-CD+sgRNA(+251) , indicated as a percentage (means ± s.e.m). Multiple unpaired t-test (A,B,C)

Supplementary Figure 8

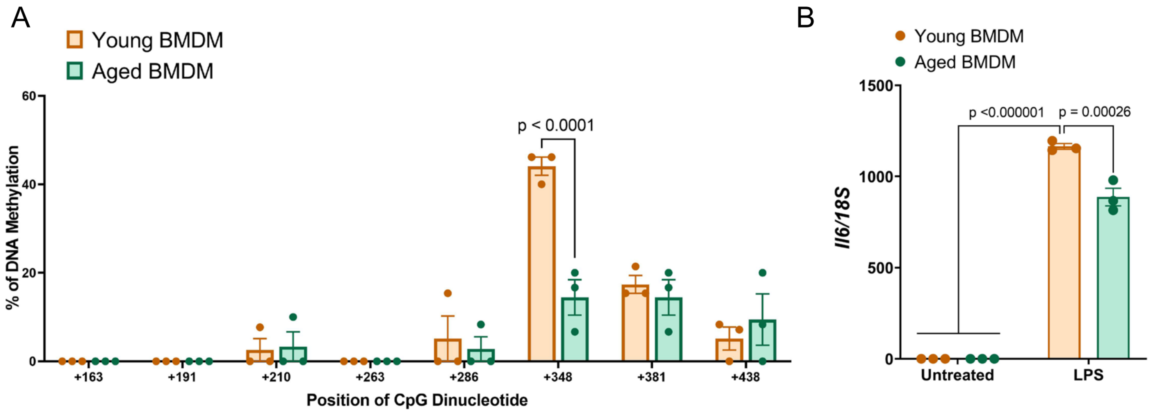

Figure S8. DNA methylation in the *Il6* locus and *Il6* expression in BMDMs, Related to Figure 6. **(A)** The methylation profile of BMDMs from young and aged mice. **(B)** The *Il6* gene expression of BMDMs from young and aged mice in the untreated or LPS-treated condition, quantified by qRT-PCR (mean ± s.e.m; $n = 3$). Multiple unpaired t-test (A) or two-way ANOVA with Tukey's multiple comparison test (B).

Supplementary Figure 9

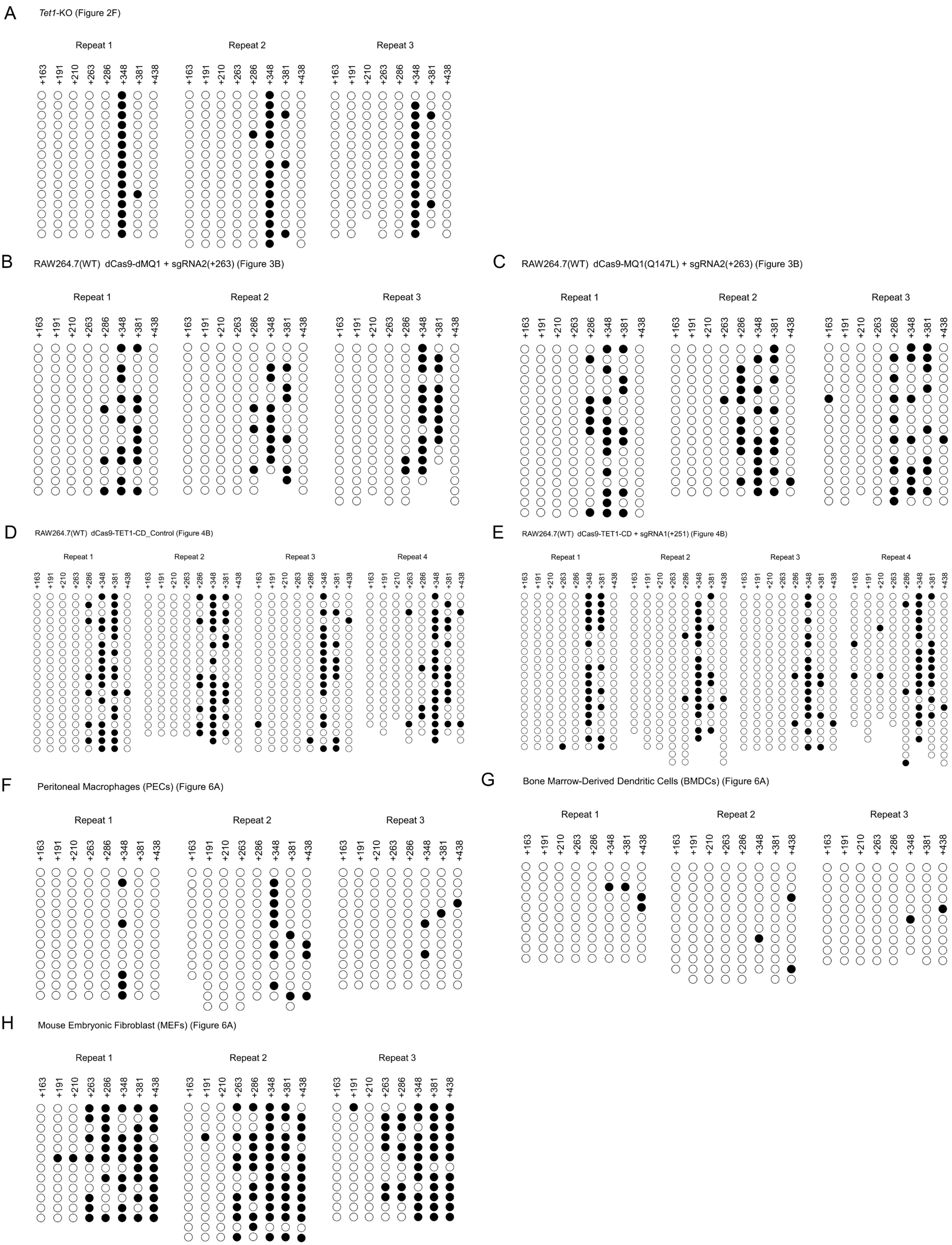

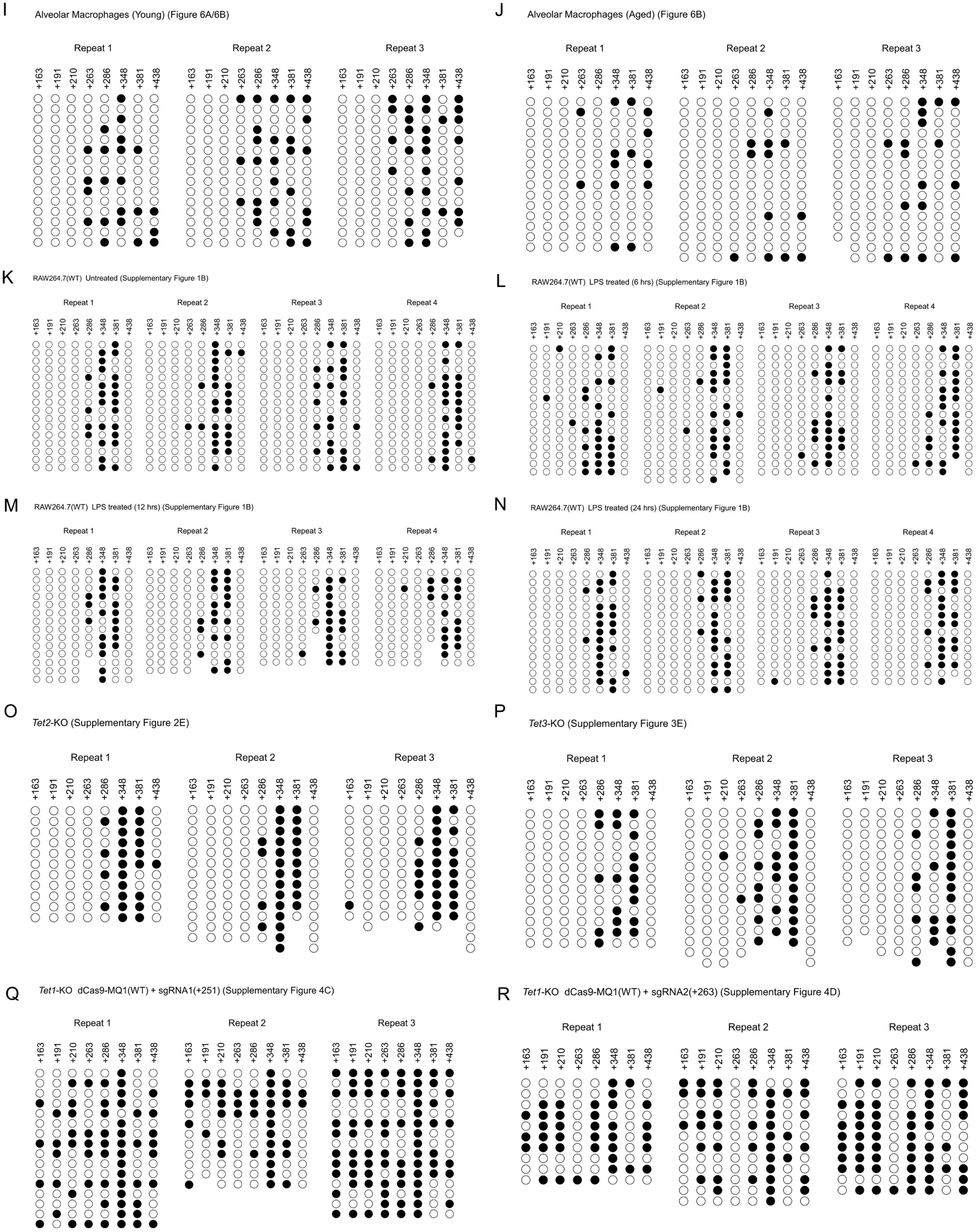
I
Alveolar Macrophages (Young) (Figure 6A/6B)
Repeat 1
Repeat 2
Repeat 3
+163 +191 +210 +263 +286 +348 +381 +438
J
Alveolar Macrophages (Aged) (Figure 6B)
Repeat 1
Repeat 2
Repeat 3
+163 +191 +210 +263 +286 +348 +381 +438
K
RAW264.7(WT) Untreated (Supplementary Figure 1B)
Repeat 1
Repeat 2
Repeat 3
Repeat 4
+163 +191 +210 +263 +286 +348 +381 +438
L
RAW264.7(WT) LPS treated (6 hrs) (Supplementary Figure 1B)
Repeat 1
Repeat 2
Repeat 3
Repeat 4
+163 +191 +210 +263 +286 +348 +381 +438
M
RAW264.7(WT) LPS treated (12 hrs) (Supplementary Figure 1B)
Repeat 1
Repeat 2
Repeat 3
Repeat 4
+163 +191 +210 +263 +286 +348 +381 +438
N
RAW264.7(WT) LPS treated (24 hrs) (Supplementary Figure 1B)
Repeat 1
Repeat 2
Repeat 3
Repeat 4
+163 +191 +210 +263 +286 +348 +381 +438
O
Tet2-KO (Supplementary Figure 2E)
Repeat 1
Repeat 2
Repeat 3
+163 +191 +210 +263 +286 +348 +381 +438
P
Tet3-KO (Supplementary Figure 3E)
Repeat 1
Repeat 2
Repeat 3
+163 +191 +210 +263 +286 +348 +381 +438
Q
Tet1-KO dCas9-MQ1(WT) + sgRNA1(+251) (Supplementary Figure 4C)
Repeat 1
Repeat 2
Repeat 3
+163 +191 +210 +263 +286 +348 +381 +438
R
Tet1-KO dCas9-MQ1(WT) + sgRNA2(+263) (Supplementary Figure 4D)
Repeat 1
Repeat 2
Repeat 3
+163 +191 +210 +263 +286 +348 +381 +438

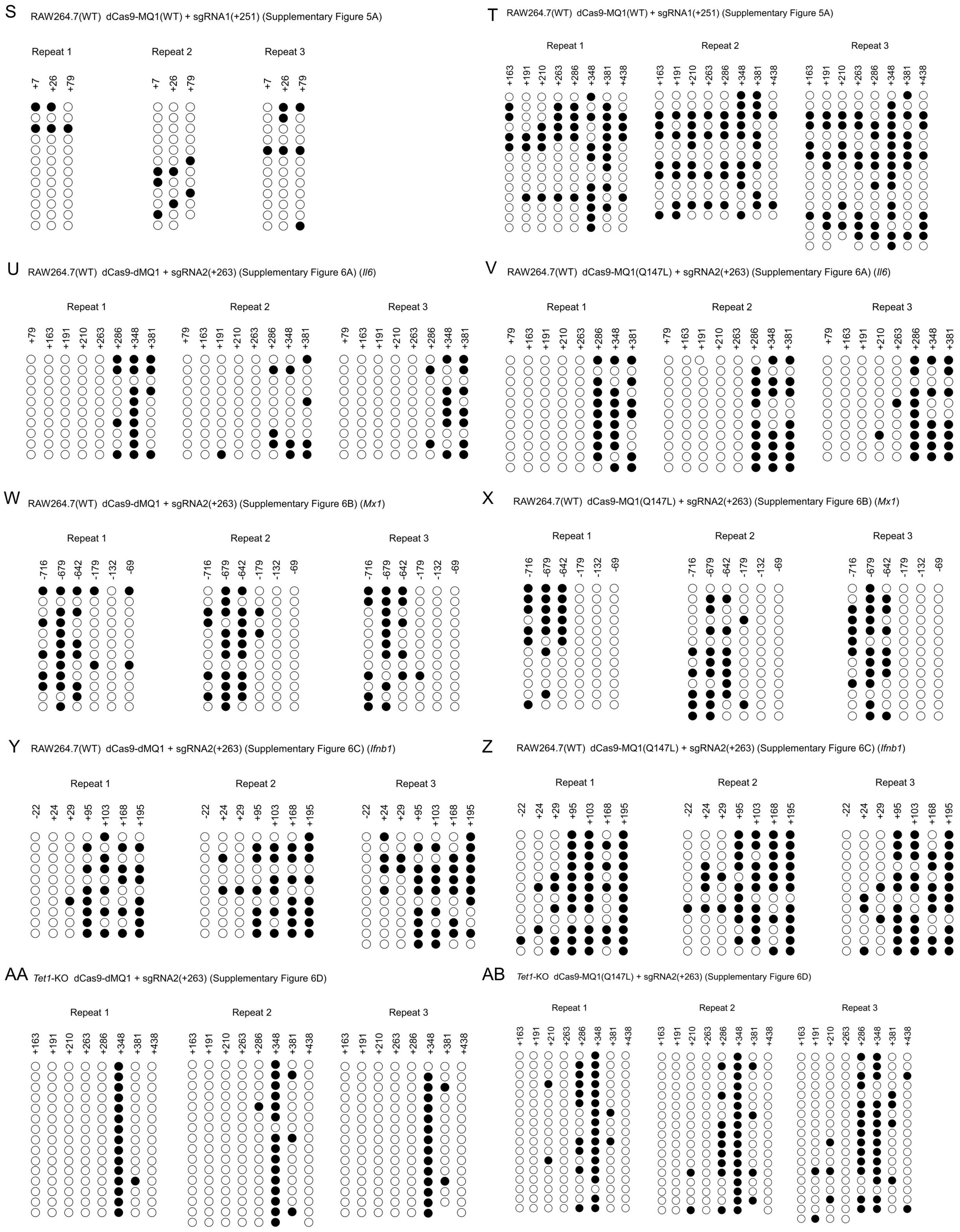
S
RAW264.7(WT) dCas9-MQ1(WT) + sgRNA1(+251) (Supplementary Figure 5A)
Repeat 1
Repeat 2
Repeat 3
+7 +26 +79
T
RAW264.7(WT) dCas9-MQ1(WT) + sgRNA1(+251) (Supplementary Figure 5A)
Repeat 1
Repeat 2
Repeat 3
+163 +191 +210 +263 +286 +348 +381 +438
U
RAW264.7(WT) dCas9-dMQ1 + sgRNA2(+263) (Supplementary Figure 6A) (*Il6*)
Repeat 1
Repeat 2
Repeat 3
+79 +163 +191 +210 +263 +286 +348 +381
V
RAW264.7(WT) dCas9-MQ1(Q147L) + sgRNA2(+263) (Supplementary Figure 6A) (*Il6*)
Repeat 1
Repeat 2
Repeat 3
+79 +163 +191 +210 +263 +286 +348 +381
W
RAW264.7(WT) dCas9-dMQ1 + sgRNA2(+263) (Supplementary Figure 6B) (*Mx1*)
Repeat 1
Repeat 2
Repeat 3
-716 -679 -642 -179 -132 -69
X
RAW264.7(WT) dCas9-MQ1(Q147L) + sgRNA2(+263) (Supplementary Figure 6B) (*Mx1*)
Repeat 1
Repeat 2
Repeat 3
-716 -679 -642 -179 -132 -69
Y
RAW264.7(WT) dCas9-dMQ1 + sgRNA2(+263) (Supplementary Figure 6C) (*Ifnb1*)
Repeat 1
Repeat 2
Repeat 3
-22 +24 +29 +95 +103 +168 +195
Z
RAW264.7(WT) dCas9-MQ1(Q147L) + sgRNA2(+263) (Supplementary Figure 6C) (*Ifnb1*)
Repeat 1
Repeat 2
Repeat 3
-22 +24 +29 +95 +103 +168 +195
AA
Tet1-KO dCas9-dMQ1 + sgRNA2(+263) (Supplementary Figure 6D)
Repeat 1
Repeat 2
Repeat 3
+163 +191 +210 +263 +286 +348 +381 +438
AB
Tet1-KO dCas9-MQ1(Q147L) + sgRNA2(+263) (Supplementary Figure 6D)
Repeat 1
Repeat 2
Repeat 3
+163 +191 +210 +263 +286 +348 +381 +438

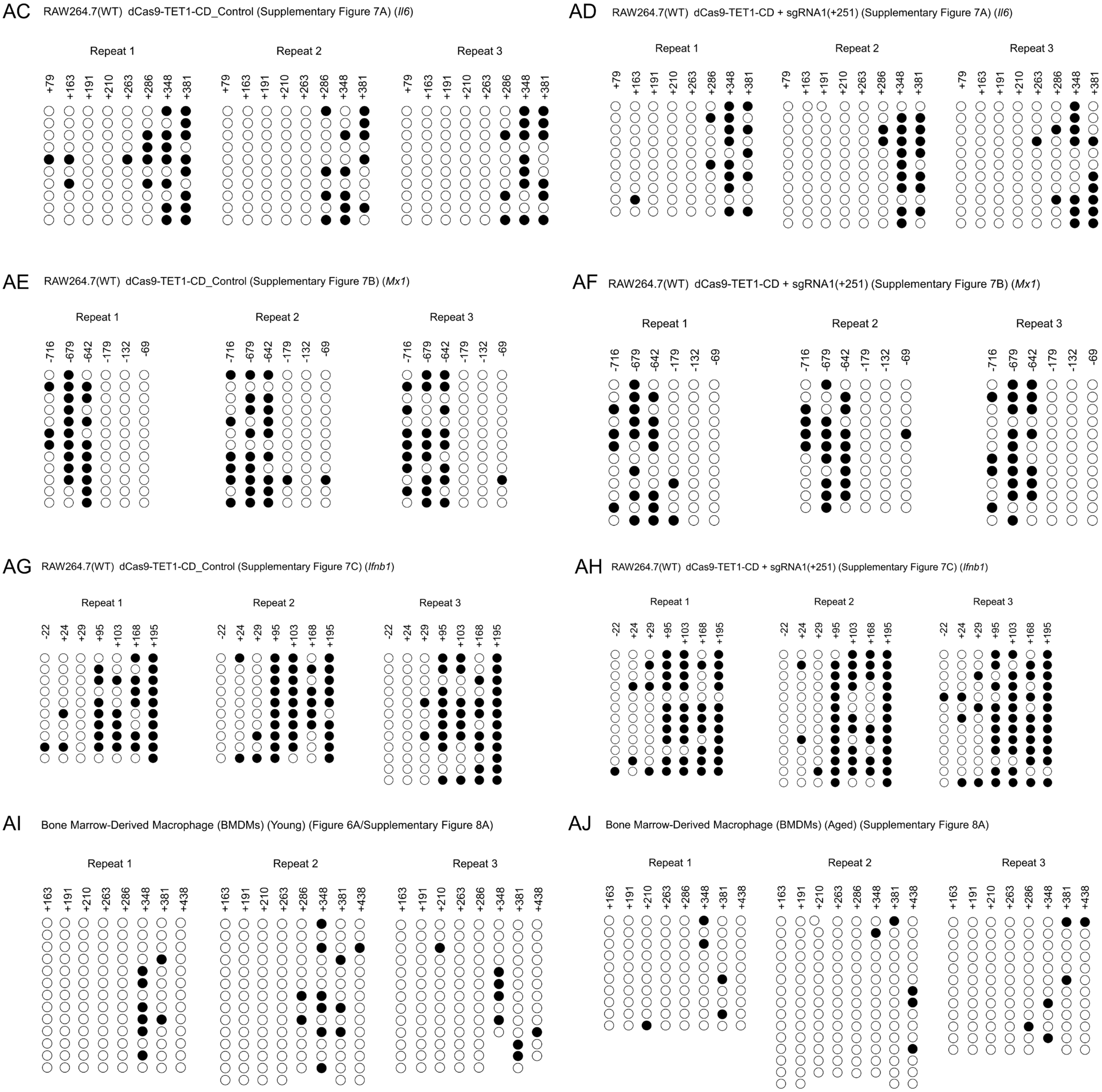

Figure S9. The DNA methylation analysis of the TIS region in the *Il6* locus in respective experiment, Related to Figure 1-4 and 6. **(A-AJ)** The DNA methylation analysis of the TIS region in the murine *Il6* locus in respective experiment. The bisulfite sequencing analysis for every experiment was repeated for three or four times. The numbers indicate the location of CpG dinucleotides at the TIS region. Open and filled circles indicate nonmethylated and methylated CpG's, respectively.

Table S2

Name	Target sequence	PAM	Strand	Mismatch 1	Mismatch 2	Mismatch 3
sgRNA for *Tet1*-KO	GCCATGAGTGTCACCACCTC	CGG	+	0	3	8
sgRNA for *Tet2*-KO	CTCACGAAGTACGTTTAAGC	CCC	-	0	0	0
sgRNA for *Tet3*-KO	ATTTGCACCTAGTCCCTCCG	GGG	+	0	0	0
sgRNA for CpG+48 deletion	TATACCACTTCACAAGTCGG	AGG	+	0	1	1
sgRNA1(+251)	TGCCTTCTTGGGACTGATGC	TGG	+	0	1	22
sgRNA2(+263)	ACTGATGCTGGTGACAACCA	CGG	+	0	2	17

Table S2. The summary of mismatch number for sgRNAs used in this study, Related to Figure 3,4 and 5. The information is obtained from https://chopchop.cbu.uib.no.

The summary of mismatch number for sgRNAs used in this study. The information is obtained from https://chopchop.cbu.uib.no.
